# SVInterpreter: A Comprehensive Topologically Associated Domain-Based Clinical Outcome Prediction Tool for Balanced and Unbalanced Structural Variants

**DOI:** 10.3389/fgene.2021.757170

**Published:** 2021-12-01

**Authors:** Joana Fino, Bárbara Marques, Zirui Dong, Dezső David

**Affiliations:** ^1^ Department of Human Genetics, National Health Institute Doutor Ricardo Jorge, Lisbon, Portugal; ^2^ Department of Obstetrics and Gynaecology, The Chinese University of Hong Kong, Hong Kong, China; ^3^ Shenzhen Research Institute, The Chinese University of Hong Kong, Shenzhen, China; ^4^ Hong Kong Hub of Pediatric Excellence, The Chinese University of Hong Kong, Hong Kong, China

**Keywords:** SVInterpreter, bioinformatic web-tool, clinical outcome prediction, balanced structural variants, copy number variants, topologically associated domains, phenotypic comparison

## Abstract

With the advent of genomic sequencing, a number of balanced and unbalanced structural variants (SVs) can be detected per individual. Mainly due to incompleteness and the scattered nature of the available annotation data of the human genome, manual interpretation of the SV’s clinical significance is laborious and cumbersome. Since bioinformatic tools developed for this task are limited, a comprehensive tool to assist clinical outcome prediction of SVs is warranted. Herein, we present *SVInterpreter,* a free Web application, which analyzes both balanced and unbalanced SVs using topologically associated domains (TADs) as genome units. Among others, gene-associated data (as function and dosage sensitivity), phenotype similarity scores, and copy number variants (CNVs) scoring metrics are retrieved for an informed SV interpretation. For evaluation, we retrospectively applied SVInterpreter to 97 balanced (translocations and inversions) and 125 unbalanced (deletions, duplications, and insertions) previously published SVs, and 145 SVs identified from 20 clinical samples. Our results showed the ability of SVInterpreter to support the evaluation of SVs by (1) confirming more than half of the predictions of the original studies, (2) decreasing 40% of the variants of uncertain significance, and (3) indicating several potential position effect events. To our knowledge, SVInterpreter is the most comprehensive TAD-based tool to identify the possible disease-causing candidate genes and to assist prediction of the clinical outcome of SVs. SVInterpreter is available at http://dgrctools-insa.min-saude.pt/cgi-bin/SVInterpreter.py.

## Introduction

Structural variants (SVs) are a class of genomic alterations that include balanced (translocations and inversions) and unbalanced (deletions, duplications, and insertions), as well as complex (cx) SVs (Collins et al., 2017). Currently, genome sequencing technologies allow a broader view of genomic variation. Nevertheless, technical issues, as breakpoints located in low complexity sequence regions that defy the bioinformatic mapping and detection tools capability, still hinder the identification of SVs (Guan and Sung, 2016).

Determining the phenotypic consequences of SVs is challenging. The diversity of its size, genomic content, location, and the intricacy of cxSV make these difficult to interpret, especially considering that they can impinge functional elements located not only within but also outside the affected genomic region (Weischenfeldt et al., 2013). Indeed, SVs alter the genome architecture of the affected regions and have a high probability of changing the position of regulatory elements, known as position effect, which may result in altered gene regulation (Spielmann et al., 2018). Previous studies showed the importance of 3D genome architecture on gene regulation, and how topologically associated domain (TAD) disruption and modification can lead to phenotypic consequences, including the alteration of chromatin loops that are recurrently associated with enhancer–promoter interaction (Lupiáñez et al., 2015; Spielmann et al., 2018)

Therefore, considering the complexity of mechanisms that can link a SV to human disease, the large number of variants identified per individual, and the substantial revision of dispersed data that this entails, ascertainment of SV pathogenicity is a daunting task (Smedley and Robinson, 2015; Zitnik et al., 2019). Furthermore, scarce integration of the available human genome annotation resources and databases also hampers clinical impact prediction of the identified variants (Lindblom and Robinson, 2011).

To date, a number of tools have been shown to tackle the role of unbalanced SVs or copy number variants (CNVs) in human diseases. Tools such as *StrVCTVRE* and *SVscore* focus on a single genomic feature to classify CNVs, as overlap with exons of important genes and precomputed pathogenicity scores of affected single nucleotide polymorphisms, respectively (Ganel et al., 2017; Sharo et al., 2020 [preprint]). *ClinTAD* provides annotation based on TAD context of each CNV, and a possible phenotypic overlap (Spector and Wiita, 2019), whereas *SVFX* uses artificial intelligence approach, based on genomic, epigenomic, and conservation features (Kumar et al., 2020).

For SVs, *AnnotSV* collects clinically relevant information on the genomic elements directly affected by breakpoints (Geoffroy et al., 2018) and *position_effect* predicts genes affected by position effects due to balanced chromosomal abnormality (BCA) breakpoints (Zepeda-Mendoza et al., 2017).

To assist the evaluation of balanced and unbalanced SVs, we previously published two useful bioinformatic tools: *TAD-GConTool* and *CNV-ConTool*. TAD-GConTool automatically defines the regions for following analysis, based on TADs affected by the breakpoints, and retrieves relevant information, whereas CNV-ConTool performs an overlap search against curated CNV databases (David et al., 2020). However, they are still limited in their scope.

Here, we present a more comprehensive tool, *SVInterpreter*, which combines the strengths of our previously published tools, with new features, to retrieve a ready-to-use data table. SVInterpreter gathers the information using breakpoints or genomic positions of balanced or unbalanced SVs, highlighting the relevant data for variant evaluation. Additionally, it performs similarity calculation between the proband’s Human Phenotype Ontology (HPO)-based clinical features and those from disorders reportedly associated to genes located within the defined regions (Köhler et al., 2019). Specifically, for CNVs, it performs an overlap search with reported CNVs in public databases and establishes classification scores according to the guidelines of American College of Medical Genetics and Genomics (ACMG) (Riggs et al., 2020).

To demonstrate the robustness of SVInterpreter, we retrospectively applied it to a set of 97 balanced (including 80 translocations and 17 inversions) and 125 unbalanced (5 insertions, 60 deletions, and 60 duplications) previously published SVs as well as 145 SVs identified in 20 clinical samples, by chromosomal microarray (CMA) or genome sequencing. Overall, we demonstrated the efficacy of this tool in retrieving exhaustive genome annotation data of genomic elements affected by SVs, allowing the prediction of their clinical significance.

## Methods

### Code and Data Sources

SVInterpreter is a Python-CGI developed Web application, freely available on https://dgrctools-insa.min-saude.pt/cgi-bin/SVInterpreter.py. The code is accessible at https://github.com/DGRC-PT/SVInterpreter, and can be run locally with an Apache configuration.

TAD data from 10 tissue or cell types, available at YUE Lab website^1^, were accessed for SVInterpreter. The regions bordering TADs–TAD boundaries—known to potentially restrict interactions of regulatory elements, were predicted using the Dixon pipeline (Dixon et al., 2012), whereas loops were established by *Peakachu* (Salameh et al., 2020).

For the chromosome Y, the TAD average size was calculated for each tissue or cell line, varying from 815 kb for lymphoblastoid cell line GM12878 to 1.8 Mb for bladder tissue (human genome assembly GRCh38/Hg38), and used as reference (Supplementary Table S1).

Full description of data sources used by SVInterpreter is available in Supplementary Table S2.

### Features and Functionality

SVInterpreter analyzes any type of balanced and unbalanced SVs larger than 1 kb (translocations, inversions, insertions, deletions, and duplications) and retrieves a table of compiled information to assist their interpretation. Complex SVs must be subdivided in distinct SVs and analyzed separately (Supplementary Figure S1). Optionally, the user can apply SVInterpreter to any genomic region, without specifying the SV type.

SVs can be mapped within cell- or tissue-specific TADs, using the breakpoints as signpost. In this case, by default, TADs affected by breakpoints (brTADs) are retrieved, with the possibility of including up to five additional breakpoint flanking TADs (TAD−5 to TAD+5). Alternatively, instead of a TAD based analysis, SVs can be analyzed within a genomic region defined by its genomic position (Supplementary Figure S2).

**FIGURE 1:**
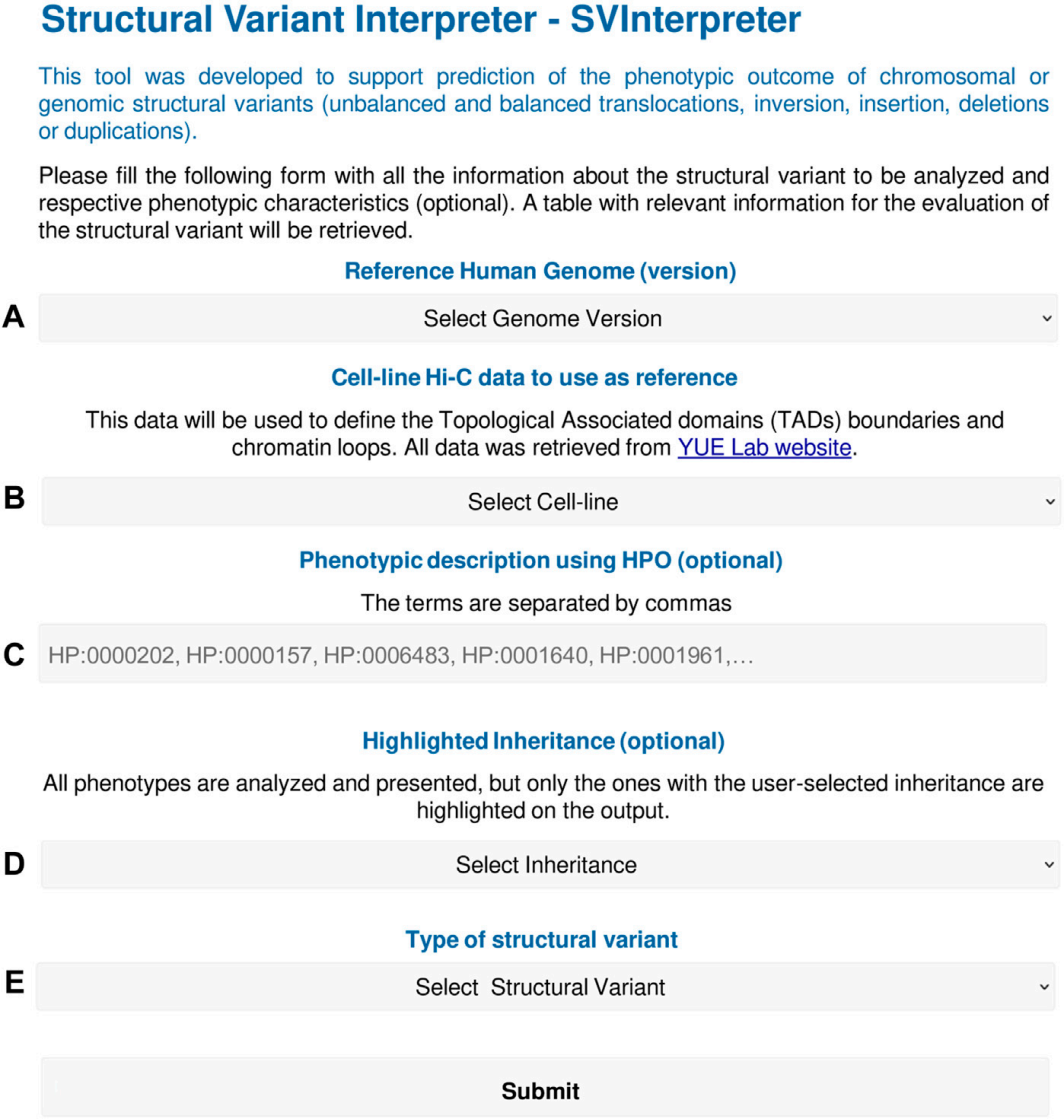
SVInterpreter input form overview. The form starts with **(A)** the selection of the human genome version (Hg19/Hg38), and then **(B)** the tissue or cell line to use as reference for TAD and loop definition. Optionally, the user can **(C)** insert the SV-associated phenotype using HPO terms or **(D)** define an inheritance of interest that will be highlighted on the output. In **(E)**, the type of SV is chosen, which will open a submenu to input the SV-specific parameters as chromosome, breakpoints, and TAD/genomic region to analyze, among others. All SVInterpreter options are shown in detail in Supplementary Figure S2.

To run SVInterpreter, a series of general parameters, such as genome version, tissue, or cell line to be used as reference for TAD and loop definition, and SVs or genomic region-specific parameters (Figure 1 and Supplementary Figure S2), are required.

From the selected specific genomic regions, SVInterpreter data were downloaded from public databases (last updated by March 31, 2021) or are automatically retrieved through an Application Programming Interface (API). From the breakpoints, all functional and non-functional genomic elements are retrieved, whereas from the remaining region, only protein-coding genes, lincRNAs, lncRNAs, functional, and non-functional genomic elements with a GTEx expression pattern are selected (Ardlie et al., 2015). Then, associated data are collected, including human disorders, cancer-specific rearrangements, phenotypes reported in animal models, genome-wide association studies (GWAS) data, and bibliography (Supplementary Table S2). The data are organized into a table, with indication of the breakpoint positions following the International System for Human Cytogenomic Nomenclature 2020 (McGowan-Jordan et al., 2020). In addition, to help visualization and interpretation of the SVs within the analyzed genomic regions, links to UCSC genome browser are made available on the output table. In this UCSC genome browser session, the selected genomic region is depicted, highlighting the SV (breakpoint or deleted/duplicated region). Native UCSC genome browser tracks compatible with the output table are shown, together with custom tracks, including the cell line/tissue-specific TADs and chromatin loops. Further description is available in Supplementary Methods.

For CNVs, SVInterpreter offers an option of performing an overlap search between the query CNV and those curated in several public CNV databases and published datasets (Supplementary Figure S2). The overlap specifications are similar to our previously published CNV-Content Tool (David et al., 2020), which retrieves the best hit by database, with the respective overlap percentage and variant frequency. In addition to the SVInterpreter standard output table, a detailed overlap table is available for download on the output web page.

Furthermore, to facilitate the evaluation of CNVs according to the ACMG guidelines, together with the standard output, the scoring parameters, as presented on the CNV pathogenicity calculator^2^, are retrieved. SVInterpreter outputs the scores for the parameters that are possible to be established automatically and then performs an automatic calculation of the final score and their respective class (Riggs et al., 2020).

The output table(s) are written in XLSX format and made available for download. Further description of the output, a step-by-step tutorial, and an application example is available in Supplementary Methods and Supplementary Table S3.

### Phenotypic Similarity Search

Optionally, the proband’s HPO-based phenotypic features can be inputted for phenotype comparison (Köhler et al., 2019).

For this, the HPO ontology provided by the HPO.db package^3^ and the links between genes, diseases, and terms provided by R data file (RDA) are a prerequisite. Since these were deprecated, we developed in-house scripts and used the June 2021 HPO release data^4^ (Köhler et al., 2019) to create state-of-the-art HPO.db and RDA files. The scripts and guidelines are available at https://github.com/DGRC-PT/HPOSim_Helper_Scripts.

The phenotype similarity is evaluated based on phenotype similarity score (PhenSSc), maximum similarity score (MaxSSc), and *p*-value (*p*), which are calculated for each combination of inputted phenotype and Online Mendelian Inheritance in Man (OMIM)^5^ phenotype associated with functional genomic elements within the analyzed region. This is performed by *HPOSim*—getTermListSim function that calculates pairwise similarities between HPO terms, using the information content (IC) of the most informative ancestor shared by both terms (Deng et al., 2015). The IC is a numeric value associated to each term, which inversely reflects the number of diseases annotated by the term, or any of its descendent terms. That is, terms with higher ICs annotate fewer diseases, being more specific, whereas lower ICs are associated to most common terms. When comparing groups of HPO terms, the getTermListSim result is the mean of the ICs of the pairwise comparisons, and reflects the similarity between the said groups, where higher scores represent higher similarities.

For PhenSSc, the inputted clinical features and the ones associated to a disorder are compared. This score reflects the similarity between the inputted phenotypic traits and the ones used to describe the disorder.

For MaxSSc, the inputted clinical features are compared with themselves, which means that MaxSSc consists of the mean of the ICs of the inputted terms. This metric was developed by us to reflect the maximum similarity score that can be obtained from the inputted terms, and to be used in comparison with PhenSSc.

The *p*-value, which reflects the probability of obtaining the PhenSSc by random chance, was adapted from Redin et al. (2017). In sum, for each disorder that PhenSSc and MaxSSc was previously calculated, a random set of HPO terms is selected. Most importantly, this set must have the same number of terms as the input, to limit the bias. The similarity score is then calculated between this set of terms and a disorder-associated phenotype (*simulated score*). Then, this is repeated 100 (*n*) times, where each time a different set of HPO terms is selected, and a new *simulated score* is obtained. Finally, the disorder specific *p*-value is calculated as:

$$P=\frac{\sum_{i=1}^{n}\left[simulatedscore_{i}\geq PhenSSc\right]}{n}$$ (1)

Phenotypes mainly composed of terms common in a wide range of disorders, as global developmental delay, or intellectual disability (with 1,386 and 1,619 associated OMIM disorders^4^, respectively), can present high PhenSSc, close to MaxSSc, and a high *p*-value as well. In these cases, the high *p*-value reflects the high probability of the phenotype to overlap by chance, warning for the limited significance of PhenSSc. Hence, ideally, the PhenSSc should be close to MaxSSc and present a *p*-value as close to zero as possible.

### SVs and Clinical Cases

For retrospective analysis, 97 and 125 previously published and unpublished balanced (translocations, inversions) and unbalanced (insertions, deletions and duplications) SVs were selected, respectively (Table 1; Supplementary Table S5) (David et al., 2003, 2009, 2015, 2018, 2020; Redin et al., 2017; Riggs et al., 2020). Of note, about half of those published by Redin et al. (2017) were previously analyzed by Zepeda-Mendoza et al. (2017), with the position_effect^6^ tool, for identification of additional candidate genes.

**TABLE 1:** SVs analyzed with SVInterpreter.

	Retrospectively reevaluated SVs			Clinical cases (PND; PN)^d^		Total by SV (retr. SVs/Clin. Cases)
	David et al.^a^	Redin et al.^b^	Riggs et al.^c^	Microarray (9; 6)^e^	liGS (0; 5)	
Translocation	9	71	0	0; 0	0; 2	80 / 2
Inversion	2	15	0	0; 0	0; 9	17/9
Deletion	2	0	58	26; 24	0; 24	60/74
Duplication	4	0	56	19; 21	0; 5	60/45
Insertion	4	1	0	0; 0	0; 13	5/13
cxSVs	0	0	0	0; 0	0; 2	0/2
Total by Publication	21	87	114	45; 45	0; 6	222/145

^a^ David et al., 2003, 2009, 2015, 2018, 2020

^b^ Redin et al., 2017

^c^ Riggs et al., 2020

^d^PND, Prenatal diagnosis; PN, Postnatal diagnosis

e PN diagnosis performed by Cytoscan HD with microarray; PND performed by Cytoscan 750K.

For effectiveness evaluation in clinical setting, nine prenatal cases (three without associated ultrasound abnormalities, four with isolated increased nuchal translucency, one with limb abnormalities, and another with multisystemic traits) and 11 postnatal cases (with isolated organ-specific or complex multisystem disorders) were used (Table 1; Supplementary Table S5). These were randomly selected among those referral for clinical diagnosis, of which genomic variants were identified by CytoScan 750K (nine cases), CytoScan HD microarrays (six cases), and long-insert genomic sequencing (liGS) (five cases). Microarray and liGS analysis was carried out as previously described (David et al., 2018, 2020).

### Criteria for SV Interpretation and Clinical Prediction

The microarray data were processed using Chromosome Analysis Suite 4.2.0.80 with NetAffx 20200828 (GRCh37/Hg19) and with the detection criteria of, at least, 15 probes within 35 kb for gains and losses. Selected SVs were manually interpreted based on the following criteria: absence/presence of OMIM genes, their association with autosomal dominant (AD) or recessive (AR) disorders, disruption of genes by the breakpoints, haploinsufficiency/triplosensitivity, and genotype/phenotype correlation (Silva et al., 2019). For this, data available at UCSC genome browser^7^, Decipher^8^, ClinGen^9^, ClinVar^10^, OMIM, DGV^11^, Unique^12^, and Orphanet^13^ databases were used.

For liGS, SVs larger than 1 kb, and CNVs identified by discordant pair clustering and coverage analysis, were selected. Then, among these, novel variants and SVs overlapping a reported variant (Collins et al., 2017; Chaisson et al., 2019, Gnomad^14^) with a database frequency <1%, and affecting loss-of-function (LoF) sensitive genes [with an expected vs. observed ratio (oe) of LoF variants of <0.35] and/or associated to AD disorders, were indicated for clinical evaluation. On average, per individual, 11 SVs were selected for analysis.

Three evaluators classified the Riggs dataset (Riggs et al., 2020) of unbalanced SVs. Therefore, based on the following criteria: (1) a classification equal by at least two of the evaluators, or (2) a median classification that reflects dissimilar evaluations, we merged them into a consensus classification (Supplementary Table S5).

To allow the comparison between published predicted outcomes of SVs and SVInterpreter-based prediction, criteria for translocations, inversions, and insertions were adapted from the previously described ones (Table 2) (Redin et al., 2017; David et al., 2020). For CNVs, ACMG guidelines were applied (Riggs et al., 2020). In addition, the same genome version and reference cell line as in the original publications was used. If available, the proband’s phenotype was inputted. For variants without pre-set of reference cell line, the human embryonic stem cell was used. By default, for all types of variants, the brTAD was used as reference, with rare exceptions. For CNVs, the overlap search against all available databases with a minimum mutual overlap of 70% was applied. The full set of variants and parameters used is available at Supplementary Table S5.

**TABLE 2:** Parameters used for the classification of SVs.

Classification	Parameters (translocation, inversion, insertion)
Pathogenic	Variant affecting or encompassing genes associated with dominant developmental disorders
Likely Pathogenic	Variant affecting or encompassing genes with a pli ≥ 0.9 not associated with disease
	Or Breakpoint located near a candidate gene associated with AD developmental disorders in a subject showing significant phenotype overlap with the referred disorder and predicted to impact long-range regulatory interactions
Variant of unknown significance (VUS)	All other variants not fitting Pathogenic, Likely Pathogenic, Likely Benign, and Benign parameters
Likely Benign	Variant affecting or encompassing genes only associated with AR disorder
	And No other data that support at least a partial overlap between the proband’s phenotype and the affected genomic region
Benign	Variant not affecting or encompassing any genes
	And No human pathology reported to be associated with genomic elements localized within the disrupted TAD or no other data that support at least the partial overlap between the proband’s phenotype and the affected genomic region

## Results

### Retrospective Reevaluation of Published SVs

For retrospective analysis, 97 balanced and 125 unbalanced previously published SVs were reevaluated (Table 1; Supplementary Table S5). With the exception of chromosome 21, SVs are distributed regularly along the genome, with an average of 12 rearrangements per chromosome. Nevertheless, the larger number of translocations (*n* = 15), inversions (*n* = 5), and insertions (*n* = 3) involved chromosome 1, chromosomes 2 and X, and chromosome 3, respectively (Supplementary Table S4).

**FIGURE 2:**
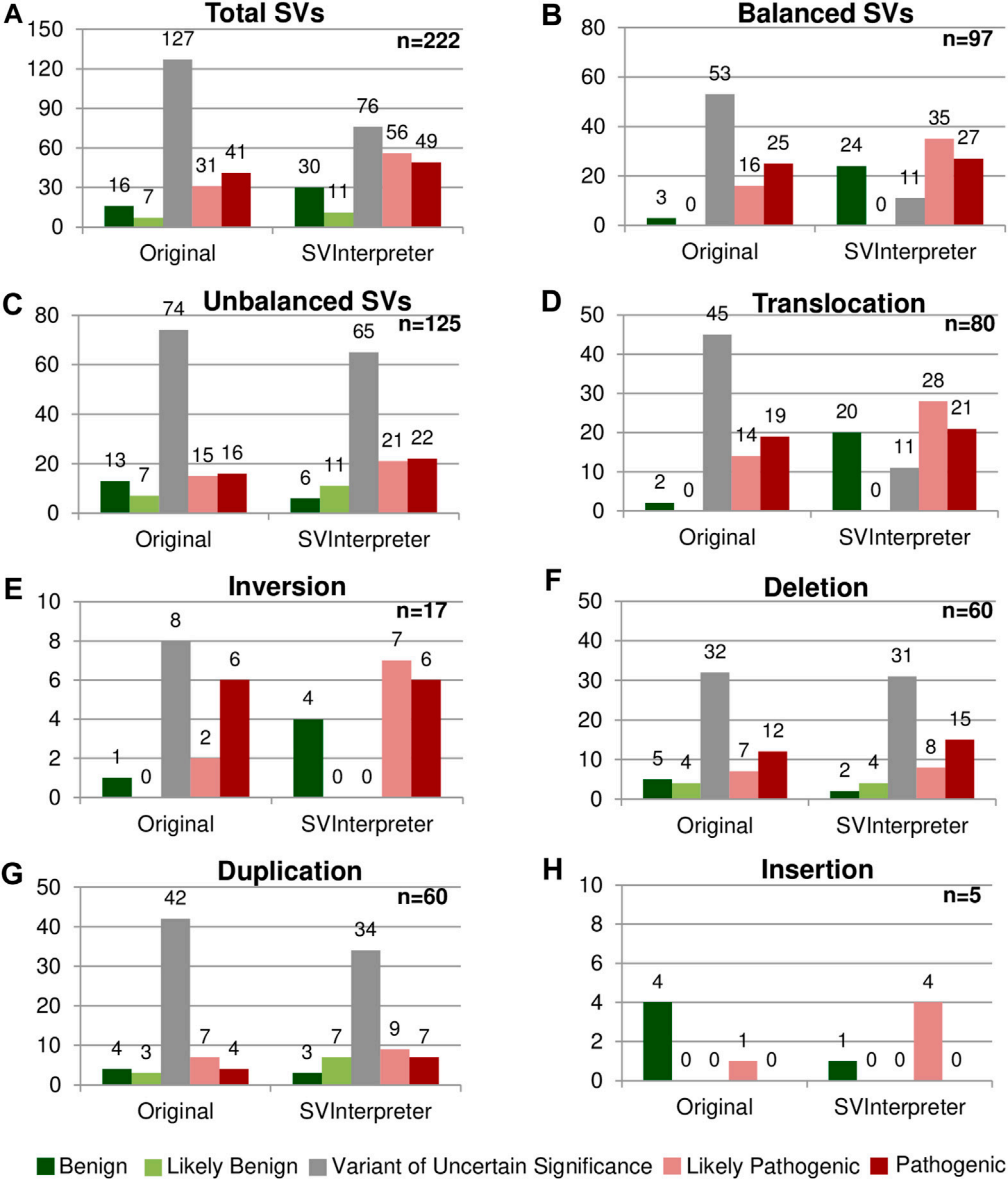
Comparison between the original and the SVInterpreter-based clinical outcome prediction. Each graphic presents the comparison between the original classification, and tool-based clinical outcome prediction for **(A)** total of SVs, **(B)** balanced SVs, **(C)** unbalanced SVs, **(D)** translocations, **(E)** inversions, **(F)** deletions, **(G)** duplications, and **(H)** insertions. Bars are color-coded, according to the clinical outcome prediction, as benign (dark green), likely benign (light green), VUS (gray), likely pathogenic (light red), and pathogenic (dark red). Number of variants is shown above the bars.

These variants were reevaluated by SVInterpreter, and based on its retrieved data, their clinical outcome was predicted according to the established parameters (Figure 2; Supplementary Table S5).

The first level of analysis involves functional and non-functional genomic elements localized within the brTADs and their annotation data, which is usually sufficient to evaluate a SV. For clinical outcome prediction of a gene disruption, SVInterpreter retrieves gene-specific annotation data such as the LoF sensitivity, Genomics England PanelApp^15^ data, its association with disorders and respective phenotypic overlap, animal model data, gene expression patters, and GWAS data.

Concomitantly, the disruption of major genes by *de novo* BCA breakpoints leading to major AD developmental disorders, as retrieved by SVInterpreter, indicated the pathogenicity of *ANKRD11* (OMIM *611192; proband DGRC0016) and *WDR26* (OMIM *617424; proband DGRC0025) (David et al., 2020). In the abovementioned cases, the calculated similarity between the inputted phenotypes and of gene-associated disorders localized within the analyzed regions played a major part on the interpretation, where *ANKRD11* PhenSSc was 2.64 (*p* = 0.02; MaxSSc = 4.01) and *WDR26* PhenSSc was 2.31 (*p* = 0.02; MaxSSc = 2.91).

If the full extent of the clinical features cannot be explained by disruption or misregulation of a candidate gene, or the breakpoint is within an intergenic region, in search for potential position effect, annotation data of all genomic elements within a brTAD must be evaluated. Several data retrieved by SVInterpreter can suggest position effect events. In addition to the phenotypic overlap and expression pattern, disruption of chromatin loops and GeneHancer clusters of interactions are important signs for possible position effect (Gloss and Dinger, 2018).

DGAP131 t(1;5)(p31;q33)dn, was originally classified by Redin et al. (2017) as variant of uncertain significance (VUS). SVInterpreter showed *MEF2C*’s (OMIM *600662) GeneHancer cluster of interactions and 10 of its 14 chromatin loops disrupted by the chromosome 5 breakpoint. The PhenSSc of 1.82 (*p* = 0.02; MaxSSc = 3.1) corroborated the proposed position effect. Likewise, *MEF2C* was indicated as a potential candidate gene by Zepeda-Mendoza et al. (2017).

Then, if the protein coding genes or functional genomic elements localized within the brTADs are insufficient to explain the observed phenotype, additional upstream (–1 to –5) and downstream (+1 to +5) flanking TADs are analyzed.

Accordingly, the t(2;11)(q14.2;q14.2) breakpoints reported in proband DGRC0001 were located in intergenic regions, and no gene at the brTADs, capable of explaining the verified phenotype, was found. At TAD+1, SVInterpreter shows that the GeneHancer cluster of interactions of the proposed candidate gene *GLI2* (OMIM * 165230) was disrupted by the 2q14.2 breakpoint, confirming the previously proposed position effect (David et al., 2009). Furthermore, the involvement of *GLI2* was reinforced by its PhenSSc of 1.33 (*p* = 0.3; MaxSSc 4.2), with disorders OMIM#615849 and OMIM#610829. Ergo, the translocation was predicted to be likely pathogenic and confirmed the published assertion of the involvement of GLI2 (David et al., 2009).

Furthermore, DGAP107 t(Y;3)(p11.2;p12.3)dn, reported by Redin et al. (2017), presents, among others, neurological defects, urinary tract, and genital abnormalities. They originally classified the SV as potentially pathogenic, due to the disruption of *ROBO2* (OMIM *602431). By assessing the associated disorder (OMIM #610878), we realized that *ROB O 2* only explained the urinary tract defects (PhenSSc = 1.12; *p* = 0.08; MaxSSc 1.48). However, SVInterpreter brTAD analysis suggested a position effect on *PCDH11Y* (OMIM * 400022), which had its GeneHancer cluster of interactions disrupted. The gene function, expression pattern, and animal model data suggest its role in the development of the nervous system, and therefore may explain the neurological defects observed in the proband. Besides, Zepeda-Mendoza et al. (2017) also indicate *SRY* (OMIM *480000), located at TAD-3, as a candidate gene due to the overlap with the genital abnormalities.

The overlap search of query CNVs in public database data and the automatic ACMG scoring showed to be of utmost utility, since it can clarify immediately the potential significance of deletions and duplications, even in cases where the genomic data are scarce. As such, a 374-kb deletion, arr[GRCh37]10q22.3(81,603,169_81,976,925)x1, in case CK without associated phenotype, was classified by Riggs et al. (2020) as VUS. According to SVInterpreter, the CNV deleted five genes that were not associated to phenotype or reported to be haploinsufficient. The CNV had 100% overlap with a likely benign ClinGen deletion (nsv3896137), and according to its ACMG CNV score of −0.9, the deletion was classified as likely benign.

Overall, more than half (57.2%) of the reevaluated SVs (45 translocations, 8 inversions, 32 deletions, and 42 duplications) were originally classified as VUS, whereas only 10.4% (23) were classified of benign and likely benign (Figures 2A–C). SVInterpreter-based reevaluation of published SVs provided a consistent finding with the original studies on 62.6% of all SVs (39 translocations, 9 inversions, 44 deletions, 45 duplications, and 2 insertions) (Supplementary Table S5). Comparatively with the original classification, the number of variants predicted as VUS decreased by 40% (from 127 to 76) (Figure 2A). For balanced SVs, SVInterpreter-based interpretation led to the reevaluation of 81.1% of the original VUS as pathogenic (9.4%), likely pathogenic (32.1%), and benign (39.6%) (Figures 2B,D,E). In addition, position effect events identified by SVInterpreter sustained the categorization of 30.2% of the potentially pathogenic balanced SVs (Supplementary Table S5). For deletions, the differences between published and tool-based prediction were minor, with similar results obtained by both (Figure 2F). Differently, 19% of the duplications categorized the VUS transited to another category, whereas only three insertions were reclassified from benign to likely pathogenic (Figures 2G,H).

**FIGURE 3:**
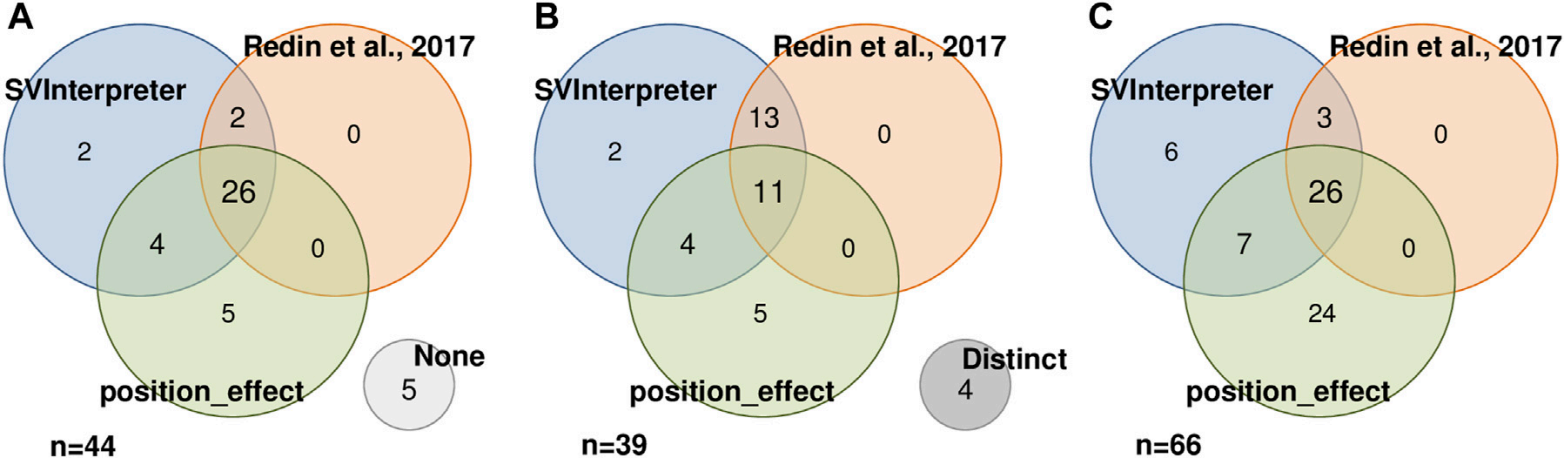
Result comparison of 44 SVs analyzed by SVInterpreter (blue), Redin et al. (2017) (orange), and position_effect (green), in three different perspectives. **(A)** SVs with associated candidate genes, including five SVs with no candidate gene identified by any of the approaches (“None”, light gray). **(B)** Similarity between the sets of candidate genes identified by SV, including four SVs where the retrieved candidate genes were different in the three approaches (“Distinct”, dark gray). **(C)** Intersection between the identified candidate genes. For **(A)** and **(B)**, the numbers inside the circles correspond to the number of SVs, while in **(C)**, the numbers represent the number of candidate genes.

To assess the position effect on distal genes and their contribution on the observed phenotypes, from the 87 balanced SVs published by Redin et al. (2017) and reevaluated by us, Zepeda-Mendoza et al. (2017) also analyzed 44 (Figure 3A). Similar candidate genes were identified in 11 of the SVs (Figure 3B), whereas in 5, neither of them proposed a candidate gene (Figure 3A). SVInterpreter and position_effect identified the same candidate genes for two originally classified VUS and two pathogenic SVs (Figure 3B; Supplementary Table S5). The position_effect tool uniquely identified 24 candidate genes in 19 SVs, where, in 14 of them, the genes were located outside the brTAD (Figure 3C; Supplementary Table S5). Based on expression, phenotypic overlap, and animal model data, SVInterpreter predicted six candidate genes not foreseen by the other two approaches, in five SVs (Figure 3C; Supplementary Table S5).

### Variant Interpretation in Clinical Setting

The effectiveness of this bioinformatic tool in a clinical setting was evaluated by comparative (manual vs. SVInterpreter-based), clinical outcome prediction of SVs identified by Cytoscan 750K microarray in nine prenatal cases, by Cytoscan HD microarray in six postnatal cases, and by liGS in five postnatal cases. Altogether, 145 variants (SVs, CNVs, and cxSVs) were analyzed (Table 1). The average number of SVs per individual, identified by genomic array, was 6, whereas for liGS, it was 289, with 44 balanced and 244 unbalanced variants. From the latter, on average, only 11 SVs (3 balanced and 8 unbalanced) were recognized to be potentially disease causing or pathogenic, and consequently selected for clinical outcome prediction (Supplementary Table S5).

Proband DGRC0004 presented a severe phenotype characterized by global developmental delay, facial dysmorphisms, and heart defects. Among other, the liGS data analysis identified a 67.3-Mb inversion inv(2)(p16.1q14.3) and a 589-kb duplication dup(2)(q21.1). Since, based on the SVInterpreter data, none of the inversion breakpoints disrupted a gene, nor any gene localized within the brTAD supported the verified phenotype, the inversion was classified as benign. Concerning the dup(2)(q21.1), although SVInterpreter identified an identical CNV in a cohort of patients with developmental delay (nsv999864) (Coe et al., 2014), the duplication has the same gene content as reported in benign SVs and did not affect triplosensitive genes, which led to its likely benign classification (ACMG CNV score = −0.9). Furthermore, none of the remaining eight clinically evaluated SVs was predicted to be likely pathogenic or pathogenic; therefore, genomic disorder was excluded in this case. Indeed, exome sequencing identified a pathogenic single-nucleotide variant within *KAT6A* (OMIM *616268) exon 18, causing AD Arboleda-Tham syndrome (OMIM #616268) (data not shown). The clinical features of this syndrome overlap that of the proband.

As CMA is the technique of choice for identification of CNVs in a clinical setting, the automatic mutual overlap search with CNV public databases and the inclusion of the ACMG scoring system is especially valuable for faster and more informed clinical outcome prediction of these.

A female in her 40s presented a dichorionic diamniotic pregnancy with an elevated risk for aneuploidy following first trimester combined screening test and normal ultrasound examination. Microarray analysis of chorionic villus sample DNA (CS750K07) identified five deletions and two duplications. By manual analysis, due to the absence of genes within the five deleted regions, these were classified as benign, whereas one of the two duplications, encompassing only a non-morbid gene, was classified as likely benign. SVInterpreter confirmed the benign and likely benign classifications, and the absence of overlapping CNVs and triplosensitive genes. In contrast, the remaining 1.1 Mb duplication at 16p13.11, arr[GRCh37]16p13.11(15,416,498_16,527,659)x3, was classified as VUS, since the CNV was overlapped by the 16p13.11 microduplication syndrome, which likely presents an incomplete penetrance and phenotypic variability. SVInterpreter identified four overlapping disorder-associated genes, *NDE1* (OMIM *609949), *MYH11* (OMIM *160745), *ABCC1* (OMIM *158343), and *ABCC6* (OMIM *603234). Although these genes are associated to AD or AR disorders, neither of them is triplosensitive or is disrupted by the breakpoints. SVInterpreter identified overlapping duplications that were reportedly classified as pathogenic (nssv15605791), likely pathogenic (nssv15149610), likely benign (nssv15159627), and VUS (nssv15159626). In addition, automatic bibliography search identified publications that described the 16p13.11 microduplication syndrome (PMID: 30287593, PMID: 23637818). Hence, in the absence of prenatal phenotype–genotype correlation, the contradictory classifications of similar duplications, and the overlap with the microduplication syndrome, we maintained the original classification of VUS.

We confirmed the reported manual clinical prediction of SVs identified in 20 individuals analyzed in a clinical setting, with marginal variability between these two approaches (Supplementary Table S5).

## Discussion

Here, we describe SVInterpreter, a web-based tool to assist the clinical outcome evaluation of balanced and unbalanced SVs. SVInterpreter assesses the regions affected by SVs, retrieves associated genome annotation data, and organizes the results in a user-friendly table. Furthermore, it scores CNVs according to ACMG criteria and assesses the overlapped variants from public databases. SVInterpeter can be used in a straightforward identification of gene disruption, evaluation of phenotypic similarities, and the indication of potential position effects within the breakpoint or flanking TADs.

As shown by retrospective analysis of the BCA cases DGRC0016 and DGRC0025 (David et al., 2020), assessment of genotype–phenotype correlation through comparison between the probands’ clinical features and of disorders caused by the disrupted genes localized within the affected genomic regions easily and quickly pointed out the pathogenicity of the analyzed variants.

Importantly, clinical setting requires tools that retrieve sufficient and adequate information to allow exclusion of the pathogenicity of SVs, in a timely fashion.

Due to the limited clinical manifestations, phenotype similarity search cannot assist in guiding the clinical outcome prediction of SVs in prenatal cases. Certainly, the availability of a dedicated fetal genotype–phenotype correlation database would further assist prenatal evaluation of SVs. Indeed, ultrasound features were absent in our prenatal sample CS750K07, making genotype–phenotype correlation practically impossible, for the 1.1-Mb duplication at 16p13.11. However, long-term follow-up would be warranted to exclude any later-onset disorder that might be associated with the SV (Halgren et al., 2018). By SVInterpreter, we were able to corroborate the manual prediction results in clinical setting, although the main advantage was essentially a more straightforward, comprehensive, and faster evaluation process.

As demonstrated by DGAP131 and DGRC0001, combination of phenotypic overlap search and identification of disrupted GeneHancer cluster of interactions and chromatin loops within the breakpoint or flanking TADs is essential for prediction of position effect events. This is true not only for breakpoints within intergenic regions where assessment of a position effect is crucial, but also for SVs where disruption of a main candidate gene is insufficient to explain the full spectrum of clinical features.

In most cases, gene disruptions or position effects within brTADs were sufficient to explain the phenotypes. Even in comparison with candidate genes uniquely identified by position_effect (Figure 3C), most of the ones located outside the brTAD showed to be associated to phenotypic traits that were already explained by genes inside the brTAD. However, in DGAP107, the full extent of associated clinical features was only resolved by a potential position effect on the third flanking TAD. This, combined with the current lack of knowledge in respect to TADs, shows the difficulty of establishing, at first hand, the region to be reviewed when evaluating an SV. This includes the arduousness of choosing, among the few, the adequate cell line or tissue to use as reference, as only recently has the TAD boundaries variability between tissues been documented (Sauerwald et al., 2020). SVIntepreter allows users to develop their own strategy to tackle this; nevertheless, we suggest to progressively increase the size of the analyzed genomic region, from the brTADs up to the fifth flanking TADs.

SVInterpreter retrieves the most comprehensive information, unraveling the role of genes not yet associated with disease. This was demonstrated by the identification of the potential candidate gene *PCDH11Y* in DGAP107, which was neglected by both Redin et al. (2017) analysis and position_effect (Zepeda-Mendoza et al., 2017).

For CNV analysis, SVInterpreter takes advantage of the resources available for unbalanced SVs. As displayed on CK and CS750K07, the overlap with database-classified CNVs and the automatic ACMG scoring made the evaluation much easier. Also, the automatic bibliography search complements the analysis, by presenting to the user a selection of publications of interest, which can provide data that eventually is unavailable on databases.

According to the features and results presented above, and especially the decrease of the previously classified VUS by 40%, we conclude that SVInterpreter alone provided enough support for assessment of the SVs. Nevertheless, we recognize that differences between Redin et al (2017) and our evaluation were affected by the fact that their classification criteria were more stringent and did not comprise benign and likely benign categories, and that additional knowledge has been acquired since their publication (El Mecky et al., 2019). Supporting this is the small number of deletions that were reclassified, since the ACMG criteria were equally used for the original and SVInterpreter-based analysis.

A major improvement of SVInterpreter was the inclusion of a function for phenotype comparison, developed mainly based on Köhler et al. (2009), Deng et al. (2015), and Redin et al. (2017). Since the phenotypic similarity scores are based on the HPO terms’ IC (Köhler et al., 2009), the score has no scale, varying with the specificity of the term, and the number of terms used for phenotype description, making it difficult to evaluate PhenSSc by itself. Therefore, MaxSSc, which reflects the upper limit of the scale for each specific set of inputted clinical features, together with the *p*-value, which measures the probability of the PhenSSc being obtained by chance, are used to interpret the PhenSSc.

Comparatively with other recent tools that support the evaluation of SVs, such as position_effect (commit: fced2c49, 13 June 2017), AnnotSV (Version 1.0, 21 December 2017) and ClinTAD (commit: 09b4925fb, 18 September 2019), SVInterpreter seems to be more comprehensive (Zepeda-Mendoza et al., 2017; Geoffroy et al., 2018; Spector and Wiita, 2019). First, SVInterpreter showed to be the one that allows more customization and adjustments, since, for example, AnnotSV and ClinTAD only work with one genome version, and ClinTAD only uses TAD boundaries of human embryonic stem cell data. Then, SVInterpreter shows a broader view of the affected regions, accounting for both gene disruption and position effects: AnnotSV is focused on the identification of genes directly affected by a breakpoint, and position_effect was designed to identify candidate genes essentially from position effect events. In regard to phenotypic comparison, as AnnotSV does not perform any, and ClinTAD is limited to the full HPO term overlap, position_effect is the only one with a similar functionality. Also, SVInterpreter is the one that retrieves the most information, including the position effect important data, GeneHancer cluster of interactions and chromatin loops, phenotypic data from DDG2P and clinGen, Gene-phenotype/disease associations in animal models, and GWAS data. Therefore, the existence of overlooked information by position_effect and AnnotSV, as shown in DGAP107, may contribute to limited results, biased candidate gene prioritization, and the need of additional resources.

Nonetheless, SVInterpreter still presents some limitations. The retrieved data are limited to the content of the available databases, which are regularly outdated with respect to the state of the art. This is currently remedied by the inclusion of the bibliographic search, but it can be improved by application of automatic text-mining systems (Luque et al., 2019). For cases of multisystemic phenotypes where more than one gene may be involved, the phenotypic overlap search could eventually be improved by adding individual phenotypic scores calculated for HPO supercategories. Additionally, SVInterpreter is prepared to analyze one variant at a time, which can be a disadvantage when dealing with complex rearrangements, or clinical cases with a large number of variants. Therefore, periodical update of this bioinformatic tool seems warranted.

The interpretation of any SV is not a straightforward task, even with the help of the right tools, since it is difficult to make sure that all factors are being considered. We do not expect SVInterpreter to change the result of the current SV evaluation, since it depends on the level of genome annotation, our current knowledge on pathological mechanisms in human disease, and, ultimately, reported data. Instead, this tool allows a well-informed and faster way to interpret SVs. Regardless of the bias given by the currently available data, attempts are being made to automate the clinical SV interpretation, which will change the current paradigm (Kumar et al., 2020). We believe that SVInterpreter, a tool to support the evaluation of balanced and unbalanced SVs, represents one more step towards this goal.

## Data Availability

The original contributions presented in the study are included in the article/Supplementary Material. Further inquiries can be directed to the corresponding authors.

## Footnotes

1
http://3dgenome.fsm.northwestern.edu/publications.html

2
https://cnvcalc.clinicalgenome.org/cnvcalc/

3
http://www2.uaem.mx/r-mirror/web/packages/HPO.db/index.html

4
https://hpo.jax.org/app/

5
https://omim.org/

6
https://github.com/ibn-salem/position_effect

7
https://genome.ucsc.edu/

8
https://decipher.sanger.ac.uk/

9
https://clinicalgenome.org/

10
https://www.ncbi.nlm.nih.gov/clinvar/

11
http://dgv.tcag.ca/dgv/app/home

12
https://rarechromo.org/

13
https://www.orpha.net/consor/cgi-bin/index.php

14
https://gnomad.broadinstitute.org/

15
https://panelapp.genomicsengland.co.uk/
