# Corrigendum: SVInterpreter: A Comprehensive Topologically Associated Domain Based Clinical Outcome Prediction Tool for Balanced and Unbalanced Structural Variants

**DOI:** 10.3389/fgene.2022.868306

**Published:** 2022-02-25

**Authors:** Joana Fino, Bárbara Marques, Zirui Dong, Dezso David

**Affiliations:** ^1^ Department of Human Genetics, National Health Institute Doutor Ricardo Jorge, Lisbon, Portugal; ^2^ Department of Obstetrics and Gynaecology, The Chinese University of Hong Kong, Hong Kong, China; ^3^ Shenzhen Research Institute, The Chinese University of Hong Kong, Shenzhen, China; ^4^ Hong Kong Hub of Pediatric Excellence, The Chinese University of Hong Kong, Hong Kong, China

**Keywords:** SVInterpreter, bioinformatic web-tool, clinical outcome prediction, balanced structural variants, copy number variants, topologically associated domains, phenotypic comparison

In the original article, there was an error. The comparison with AnnotSV was made with its first version, which is currently out of date. Any remark made on this tool should not be considered.

The versions, commits and release dates of the tools used for comparison were not indicated.

Therefore, a correction has been made to **Discussion**, paragraph 11:

“Comparatively with other recent tools that support the evaluation of SVs, such as position_effect (commit: fced2c49, 13 June 2017), AnnotSV (Version 1.0, 21 December 2017) and ClinTAD (commit: 09b4925fb, 18 September 2019), SVInterpreter seems to be more comprehensive (Zepeda-Mendoza et al., 2017; Geoffroy et al., 2018; Spector and Wiita, 2019). First, SVInterpreter showed to be the one that allows more customization and adjustments, since, for example, AnnotSV and ClinTAD only work with one genome version, and ClinTAD only uses TAD boundaries of human embryonic stem cell data. Then, SVInterpreter shows a broader view of the affected regions, accounting for both gene disruption and position effects: AnnotSV is focused on the identification of genes directly affected by a breakpoint, and position_effect was designed to identify candidate genes essentially from position effect events. In regard to phenotypic comparison, as AnnotSV does not perform any, and ClinTAD is limited to the full HPO term overlap, position_effect is the only one with a similar functionality. Also, SVInterpreter is the one that retrieves the most information, including the position effect important data, GeneHancer cluster of interactions and chromatin loops, phenotypic data from DDG2P and clinGen, Gene-phenotype/disease associations in animal models, and GWAS data. Therefore, the existence of overlooked information by position_effect and AnnotSV, as shown in DGAP107, may contribute to limited results, biased candidate gene prioritization, and the need of additional resources.”

The authors apologize for the errors and state that this does not change the scientific conclusions of the article in any way. The original article has been updated.

