# Supplementary material for: SVInterpreter: A Comprehensive Topologically Associated Domain-Based Clinical Outcome Prediction Tool for Balanced and Unbalanced Structural Variants
Source: Front Genet. 2021 Dec 1;12:757170. doi: 10.3389/fgene.2021.757170 (PMC8671832; doi:10.3389/fgene.2021.757170)
Supplement: Supplementary file 1 [file Table4.PDF]

Supplementary Table 4. Distribution of the individual SVs analyzed by chromosome

	Chromosome																							
	**1**	**2**	**3**	**4**	**5**	**6**	**7**	**8**	**9**	**10**	**11**	**12**	**13**	**14**	**15**	**16**	**17**	**18**	**19**	**20**	**21**	**22**	**X**	**Y**
**Translocation**	15	10	10	5	10	7	9	5	6	6	11	7	5	13	4	4	8	5	6	3	0	1	8	2
**Inversion**	1	5	1	1	1	1	1	0	0	0	0	0	1	0	0	0	0	0	0	0	0	0	5	0
**Deletion**	5	8	5	1	1	2	3	1	4	3	2	1	3	3	5	6	1	1	1	2	0	0	2	0
**Duplication**	1	6	3	0	3	2	2	2	3	1	2	3	3	2	4	6	4	2	2	0	0	2	7	0
**Insertion**	1	1	3	0	0	0	0	1	1	0	0	0	0	0	0	0	1	0	0	0	0	1	0	0
**Total**	**23**	**30**	**22**	**7**	**15**	**12**	**15**	**9**	**14**	**10**	**15**	**11**	**12**	**18**	**13**	**16**	**14**	**8**	**9**	**5**	**0**	**4**	**22**	**2**

Interchromosomal SVs are accounted for in both chromosomes involved in the rearrangement, while intrachromosomal SVs are only counted once.
