# Supplementary material for: SVInterpreter: A Comprehensive Topologically Associated Domain-Based Clinical Outcome Prediction Tool for Balanced and Unbalanced Structural Variants
Source: Front Genet. 2021 Dec 1;12:757170. doi: 10.3389/fgene.2021.757170 (PMC8671832; doi:10.3389/fgene.2021.757170)
Supplement: Supplementary file 2 [file Table1.PDF]

Supplementary Table 1. Average TAD size by genome version and cell line or tissue

Cell line/Tissue	Hg38 average TAD size (bp)	Hg19 average TAD size (bp)
Consensus TADs	1,796,527	1,786,131
IMR90	858,229	824,472
LCL (GM12878)	815,201	835,946
hESC	1,087,767	1,058,135
A549	1,405,466	1,411,623
Aorta tissue	1,679,636	1,690,650
Cortex tissue	1,736,794	1,337,331
Bladder tissue	1,799,711	1,672,068
Lung tissue	1,754,399	1,435,346
HUVEC	1,072,369	923,778
K562	842,166	867,120

This average TAD size values are used to define the regions to analyze on chromosome Y. For each chromosome Y breakpoint, the region to analyze will start at [breakpoint – (average TAD size/2)] and end at [breakpoint+(average TAD size/2)]
