# Supplementary material for: SVInterpreter: A Comprehensive Topologically Associated Domain-Based Clinical Outcome Prediction Tool for Balanced and Unbalanced Structural Variants
Source: Front Genet. 2021 Dec 1;12:757170. doi: 10.3389/fgene.2021.757170 (PMC8671832; doi:10.3389/fgene.2021.757170)
Supplement: Supplementary file 3 [file Table2.PDF]

Supplementary Table 2. Data sources used by SVInterpreter

Data	Description	Information used	Source	Reference
Topological Associated Domains (TADs)	TADs are functional units of the genome. They are self-interacting genomic regions, which means that DNA sequences inside the TAD physically interact with each other more frequently than with sequences outside the TAD.	The TADs are used as measure units for the output table construction, defining the interest regions to be analyzed on the context of a variant.	http://3dgenome.fsm.northwestern.edu/publications.html	Rao et al., 2014; Dixon et al., 2015; Lajoie et al., 2015; Leung et al., 2015; Schmitt et al., 2016; Li et al., 2019
Genomic elements (Ensembl)	Ensembl is a genome browser for vertebrate genomes that supports research in comparative genomics, evolution, sequence variation and transcriptional regulation.	Genomic elements located interest regions, and associated information, including orientation and synonyms.	https://www.ensembl.org/index.html	Hunt et al., 2018
Genecards	Database of human genes that provides concise genomic related information, on all known and predicted human genes.	The direct hyperlink for each gene on the database is made available.	https://www.genecards.org/	Stelzer et al., 2016
Genomics England PanelApp	PanelApp is a publicly available knowledgebase that allows virtual gene panels related to human disorders to be created, stored, and queried.	Indication of which categories the panel that contains the gene fits and the respective level of evidence.	https://panelapp.genomicsengland.co.uk/	Martin et al., 2019
Actionable genes	The American College of Medical Genetics and Genomics (ACMG) has compiled a list of 59 genes, for which specific mutations are known to be causative of disorders with defined phenotypes that are clinically actionable by an accepted intervention.	Indication if the gene is on the list of the 59 genes.	https://www.ncbi.nlm.nih.gov/clinvar/docs/acmg/	Kalia et al., 2017
OMIM	OMIM is a comprehensive, authoritative compendium of human genes and genetic phenotypes	OMIM gene ID, gene function description, associated phenotypes, inheritance and HPO phenotypic characteristics.	https://omim.org/	nd
Haploinsufficiency index (HI)	Describes a model of dominant gene action in diploid organisms, in which a single copy of the wild-type allele at a locus in heterozygous combination with a variant allele is insufficient to produce the wild-type phenotype.	HI of each gene.	https://decipher.sanger.ac.uk/about/downloads/data	Huang et al., 2010
Triplosensitivity (Triplo)	Evidence that a duplication of the genomic region leads to a specific phenotype.	Triplo of each gene.	https://dosage.clinicalgenome.org/	nd

probability loss of function (pli) and observed vs expected ratio (oe score)	Measures the tolerance of a gene to loss of function mutation.	pli and confidence interval of oe score for each gene.	https://gnomad.broadinstitute.org/	Karczewski et al., 2020
Uniprot	Comprehensive, high-quality and freely accessible resource of protein sequence and functional information	Direct link to the set of proteins obtained from each specific gene, for human and other animal models.	https://www.uniprot.org/	Bateman et al., 2021
GTEx expression	Public resource to study tissue-specific gene expression and regulation.	Top 3 expressed tissues for each genomic elements, including their expression quantification, the mean expression and total expression.	https://www.gtexportal.org/home/	Ardlie et al., 2015
Clustered interactions of GeneHancer	GeneHancer is a database of human regulatory elements (enhancers and promoters) and their inferred target genes	Region of interaction of each gene.	https://www.genecards.org/	Fishilevich et al., 2017
Chromatin Loops	Loops of interaction specific for the cell line or tissue chosen for the TADs	Regions involved on the loop, including or not genomic elements.	http://3dgenome.fsm.northwestern.edu/publications.html	Salameh et al., 2020
Developmental disorder gene to phenotype (DDG2P)	Integrates data from genes, variants, and phenotypes regarding developmental disorders.	Phenotypes associated to genomic elements, and respective classification.	https://www.ebi.ac.uk/gene2phenotype/disclaimer	Wright et al., 2015
ClinGen	CliGen evidence of the association between a gene and a phenotype	Phenotypes associated to genomic elements, and respective classification.	https://clinicalgenome.org/	Rehm et al., 2015
HPOSim similarity analysis	Calculation of similarities between groups of HPO terms.	Similarity score calculation between case's phenotype and gene-associated phenotypes. The similarity score, Maximum score and p-value is showed for each case.	https://cran.r-project.org/src/contrib/Archive/HPOSim/	Deng et al., 2015
Fusion Gene in cancer	Data about fusion genes found in different types of cancer according to the Mitelman Database and Atlas of Genetics and Cytogenetics in Oncology and Haematology	Fusion genes involving each gene, the type of cancer and the respective number of cases.	http://atlasgeneticsoncology.org/ https://mitelmandatabase.isb-cgc.org/	Huret et al., 2013

C. elegans model organism (WormBase)	WormBase contains accurate, current, accessible information concerning the genetics, genomics and biology of *C. elegans* and related nematodes.	Orthologs of the human genes identified and respective knockout phenotypic characteristics.	https://wormbase.org	Harris et al., 2020
Drosophila model organism (FlyBase)	FlyBase contains user-friendly information concerning the biology of *Drosophila*.	Orthologs of the human genes identified and respective knockout phenotypic characteristics.	https://flybase.org/	Thurmond et al., 2019
Mouse model organism (MGI)	MGI provides integrated genetic, genomic, and biological mouse data to facilitate the study of human health and disease.	Orthologs of the human genes identified and respective knockout phenotypic characteristics.	http://www.informatics.jax.org/	Eppig, 2017
Zebrafish model organism (zfin)	Database of genetic and genomic data for zebrafish (*Danio rerio*) providing a wide array of expertly curated, organized, and cross-referenced zebrafish research data.	Orthologs of the human genes identified and respective knockout phenotypic characteristics.	https://zfin.org/	Sprague et al., 2003
Infertility genes	Genes associated to infertility	Type of infertility disorder associated to the respective gene.	nd	Oud et al., 2019
Genome wide association studies (GWAS) - SNP data	Human catalog of phenotype-associated SNPs and genes	SNPs, genes and phenotypic characteristics and the level significance according to the p-value.	https://www.ebi.ac.uk/gwas/	Buniello et al., 2019
PubMed	Gene associated publications	Automatic search of the genes in the PubMed database.	https://pubmed.ncbi.nlm.nih.gov/	nd
CNV databases	Benign to Pathogenic CNVs, from several public curated databases, including DGV, 1000 genomes, ClinGen and Gnomad SV. Reference publications data is also used.	Benign to Pathogenic CNVs, percentages of overlap, frequencies, and respective location.	http://dgv.tcag.ca/dgv/app/home https://clinicalgenome.org/ https://gnomad.broadinstitute.org/	Cooper et al., 2012; Coe et al., 2014; MacDonald et al., 2014; Rehm et al., 2015; Collins et al., 2017; Chaisson et al., 2019; Karczewski et al., 2020
Marrvel	Integration of human and model organism genetic resources to facilitate functional annotation of the human genome.	Retrieving integrated data, namely the association between human genes and their respective orthologs.	http://marrvel.org/	Wang et al., 2017

nd - Not determined
