# Supplementary material for: SVInterpreter: A Comprehensive Topologically Associated Domain-Based Clinical Outcome Prediction Tool for Balanced and Unbalanced Structural Variants
Source: Front Genet. 2021 Dec 1;12:757170. doi: 10.3389/fgene.2021.757170 (PMC8671832; doi:10.3389/fgene.2021.757170)
Supplement: Supplementary file 4 [file Image2.PDF]

A

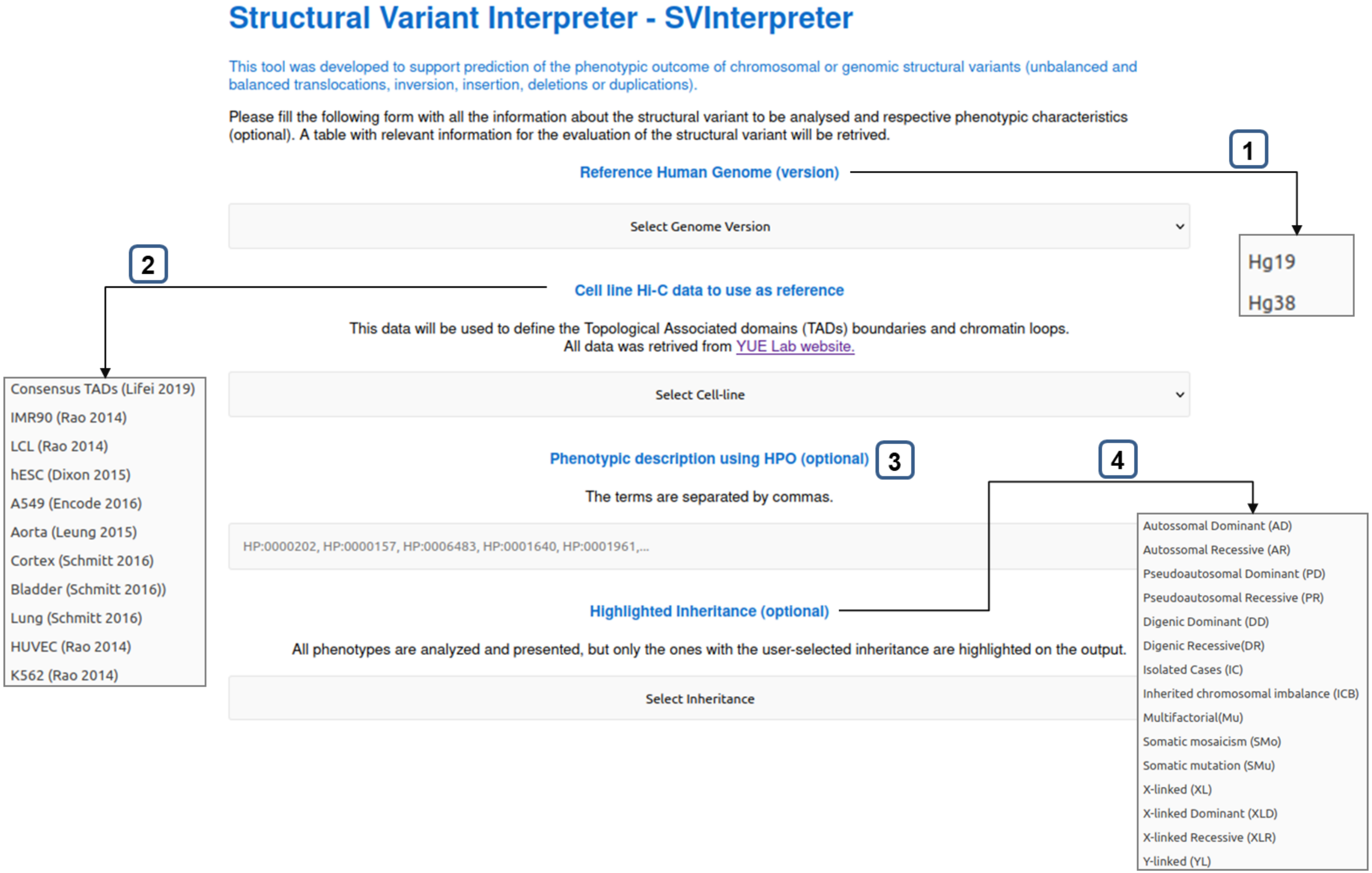

Structural Variant Interpreter - SVInterpreter
This tool was developed to support prediction of the phenotypic outcome of chromosomal or genomic structural variants (unbalanced and balanced translocations, inversion, insertion, deletions or duplications).
Please fill the following form with all the information about the structural variant to be analysed and respective phenotypic characteristics (optional). A table with relevant information for the evaluation of the structural variant will be retrived.
Reference Human Genome (version)
1
Select Genome Version
Hg19
Hg38
2
Cell line Hi-C data to use as reference
This data will be used to define the Topological Associated domains (TADs) boundaries and chromatin loops.
All data was retrived from YUE Lab website.
Consensus TADs (Lifei 2019)
IMR90 (Rao 2014)
LCL (Rao 2014)
hESC (Dixon 2015)
A549 (Encode 2016)
Aorta (Leung 2015)
Cortex (Schmitt 2016)
Bladder (Schmitt 2016))
Lung (Schmitt 2016)
HUVEC (Rao 2014)
K562 (Rao 2014)
Select Cell-line
Phenotypic description using HPO (optional)
3
4
The terms are separated by commas.
HP:0000202, HP:0000157, HP:0006483, HP:0001640, HP:0001961,...
Highlighted Inheritance (optional)
All phenotypes are analyzed and presented, but only the ones with the user-selected inheritance are highlighted on the output.
Select Inheritance
Autossomal Dominant (AD)
Autossomal Recessive (AR)
Pseudoautosomal Dominant (PD)
Pseudoautosomal Recessive (PR)
Digenic Dominant (DD)
Digenic Recessive(DR)
Isolated Cases (IC)
Inherited chromosomal imbalance (ICB)
Multifactorial(Mu)
Somatic mosaicism (SMo)
Somatic mutation (SMu)
X-linked (XL)
X-linked Dominant (XLD)
X-linked Recessive (XLR)
Y-linked (YL)

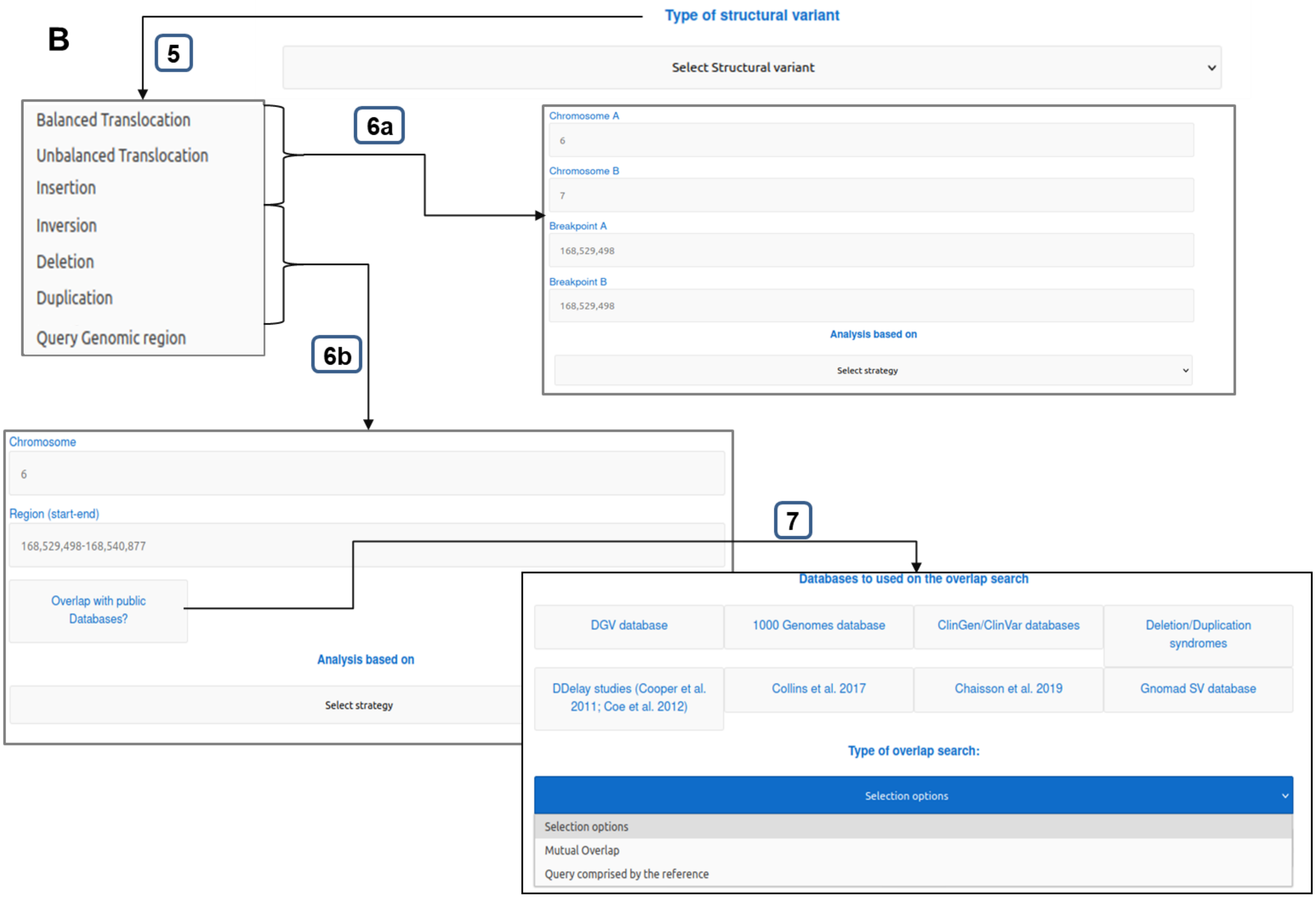

B
5
Type of structural variant
Select Structural variant
Balanced Translocation
Unbalanced Translocation
Insertion
Inversion
Deletion
Duplication
Query Genomic region
6a
Chromosome A
6
Chromosome B
7
Breakpoint A
168,529,498
Breakpoint B
168,529,498
Analysis based on
Select strategy
6b
Chromosome
6
Region (start-end)
168,529,498-168,540,877
Overlap with public Databases?
Analysis based on
Select strategy
7
Databases to used on the overlap search
DGV database
1000 Genomes database
ClinGen/ClinVar databases
Deletion/Duplication syndromes
DDelay studies (Cooper et al. 2011; Coe et al. 2012)
Collins et al. 2017
Chaisson et al. 2019
Gnomad SV database
Type of overlap search:
Selection options
Selection options
Mutual Overlap
Query comprised by the reference

C

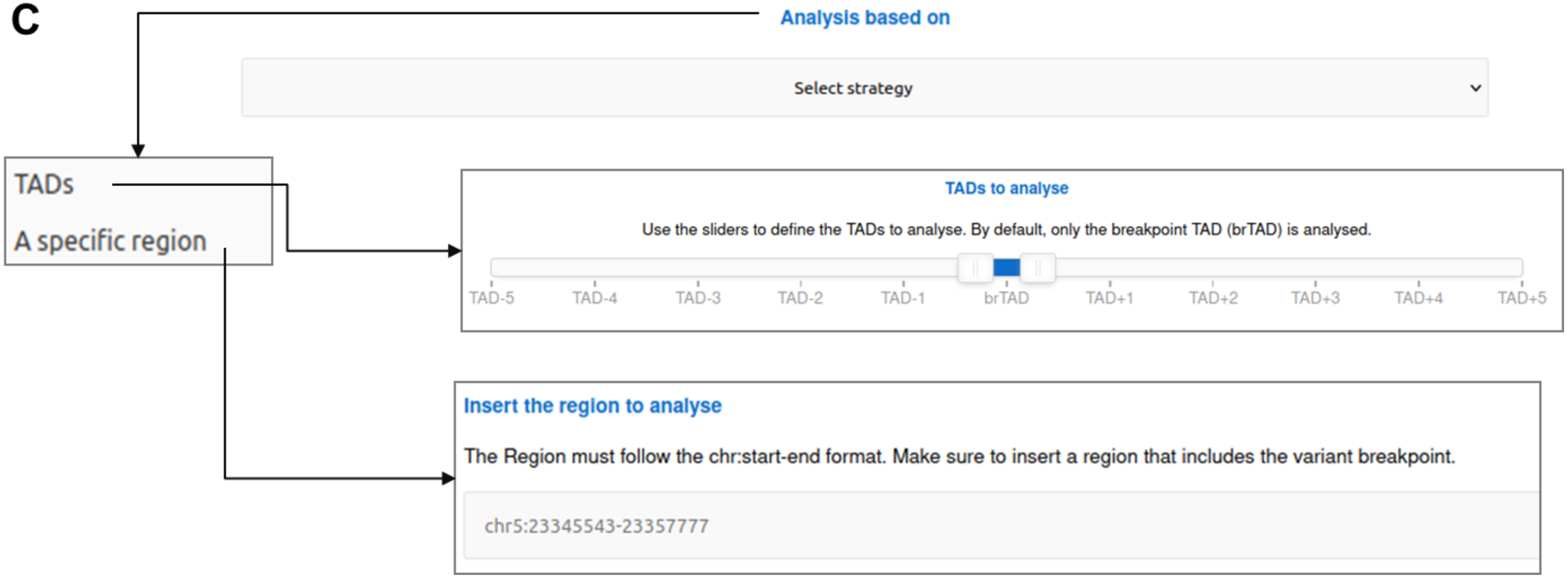

Supplementary Figure 2. SVInterpreter Input form overview. The SVInterpreter form can be divided into three parts, (**A**) the general parameters, (**B**) SV specific parameters, and (**C**) Selection of region to analyze.

(**A**) Starts with the selection of the (**1**) human genome version and the (**2**) reference cell line to be used for TAD and Loop search. Then, two optional parameters: (**3**) phenotypic description of the case in question, which must be inputted using Human Phenotype Ontology terms separated by commas; and (**4**) the selection of an inheritance of interest, where the disorders with the selected inheritance will be highlighted on the output table.

(**B**) Then, (**5**) type of variant is chosen, where each type of variant will open a slightly different form. For insertions, balanced and unbalanced translocations, the form is similar to the one showed in (**6a**), where the user sets the chromosomes and breakpoints. For deletions, duplications, and inversions a form similar to (**6b**) is showed, with the choice of CNV database overlap search, for deletions and duplications. Choosing the (**7**) CNV overlap search, the user must select which databases to use, and the type of overlap, as described in David et al., (2020).

(**C**) Lastly, the user can choose between using TADs to define the region to analyze (where the user can choose up to 5 TADs upstream (+5) or downstream (-5) the breakpoint) or set the region manually using genomic coordinates.
