# Supplementary material for: SVInterpreter: A Comprehensive Topologically Associated Domain-Based Clinical Outcome Prediction Tool for Balanced and Unbalanced Structural Variants
Source: Front Genet. 2021 Dec 1;12:757170. doi: 10.3389/fgene.2021.757170 (PMC8671832; doi:10.3389/fgene.2021.757170)
Supplement: Supplementary file 6 [file Table3.PDF]

Supplementary Table 3. **SVInterpreter output table column description**

Category	Column	Description
**Genes and intergenic regions**	Genomic elements; Pannels from PannelApp	Genes located inside the TAD; The principal symbol of the gene is presented, with the respective synonyms (if exist) inside brackets. The gene is followed by the PanelAPP associated panels and respective level of evidence, in which case, it's all presented in bold. The gene symbol has a hyperlink to the GeneCard respective page. Intergenic regions and the breakpoint locations are also presented in this column.
	Actionable Genes (MAGs)	The American College of Medical Genetics and Genomics actionable genes are presented in this column. If exists, appears in bold.
	Breakpoint location; Genome strand	If the gene is disrupted or partially deleted/duplicated, the affected region is presented here. Then the strand of the gene is presented next, as SS for sense, and AS for antisense. Breakpoint location and strand are separated by a semicolon. If information about the disruption is present, this field appears in bold and green.
	Gene ID	Gene ID on the OMIM database. The ID has a hyperlink to the respective page on the OMIM website.
	HI% ; Triplo	Indicates the Haploinsuficiency index and triplosensitivity score of the gene, separated by a semicolon. If the Haploinsuficiency index is lower than 10% or the triplosensitivity score is equal to 3, this field appears in bold and green.
	pLi; o/e score	Indicates the probability of loss of function and the observed vs expected score, both values separated by a semicolon. If the observed vs expected score is lower than 0.3, this field appears in bold and green.
	Protein entries	Direct link to Uniprot database, to a set of proteins associated to the respective gene, in the human species.
	Function	Description of the gene function, according to OMIM. The description might not be complete since this field is limited by the number of characters allowed on a XLSX cell.
	Top 3 highest TPM (Total TPM; Mean TPM)	The three most expressed tissues according to GTEx, in Transcripts per million (TPM). Besides the value of expression of each individual tissue, the total and mean expression are also presented inside brackets and separated by a semicolon.
**Clustered interactions and Loops**	Clustered interactions	The region covered by the cluster of interactions of each gene, according to GeneHancer. If the region is disrupted by the breakpoint, the text is presented in bold and green.
	Loops	Loops identified on the cell line or tissue chosen in the input form. The two genomic regions involved on the Loop are inside brackets, preceded by the genomic elements that they affect, and separated by "&&". If the loop is disrupted by the alteration, the field is presented in bold and green.
**Clinical phenotype**	Assoc. Disorder	Description of the disorder associated to the gene. This field has the direct hyperlink to OMIM, DDG2P or ClinGen, according to the source of the information.
	OMIM_ID_inh	ID of the OMIM phenotype indicated in the previous column, and the respective inheritance separated by an underscore, with hyperlink to the respective page on the OMIM website. If the inheritance matches the one chosen by the user on the input form (optional), this field appears in bold.
	DDG2P class.	Classification of the disorders described on the Assoc. Disorder column, according to DDG2P.
	ClinGen class.	Classification of the disorders described on the Assoc. Disorder column, according to ClinGen.

	PhenSSc (P; MaxSSc)	Result of the phenotype similarity search if any phenotype was inputted on the input form (optional). The first score is the similarity score between the inputted phenotype and the disorder described on the Assoc. Disorder column. Next, inside brackets, and separated by a semicolon is the p-value that reflects the probability of this score been obtained by chance and the maximum score that could be attained with the inputted phenotype description. This search is only applicable to disorders described on OMIM, with associated phenotypic description.
**Gene fusion in cancer**	Gene1 / Gene2 Cytoband	Fusion genes found in cancer. The two genes fused are separated by a bar, and followed by the cytoband of the second gene, separated by an underscore. The first gene is always the one being described in this line. The text has hyperlink to the Atlas database or the Mitleman database.
	Organ: type, nr. Cases	Organ and the type of cancer where the previous fusion gene was found, followed by the number of cases. The text has hyperlink to the Atlas database or the Mitleman database.
**Gene-phenotype/disease associations and animal models**	C.elegans	Phenotypic characteristics of the knockout results of the Orthologs of the human gene in *C.elegans*, with link to WormBase. Also a direct link to Uniprot search in *C.elegans* is provided.
	Drosophila	Phenotypic characteristics of the knockout results of the Orthologs of the human gene in fruit fly, with link to FlyBase. Also, a direct link to Uniprot search in fruit fly is provided.
	Mouse	Phenotypic characteristics of the knockout results of the Orthologs of the human gene in mouse, with link to MGI. Also, a direct link to Uniprot search in mouse is provided.
	Rat	Phenotypic characteristics of the knockout results of the Orthologs of the human gene in rat, with link to RGD. Also a direct link to Uniprot search in rat is provided.
	Zebrafish	Phenotypic characteristics of the knockout results of the Orthologs of the human gene in zebrafish, with link to Zfin. Also, a direct link to Uniprot search in zebrafish is provided.
**Infertility**	Disorder	Infertility-associated disorders, that were potentially or confirmed as associated with the gene in question. The disorder is presented in the column, followed by the type of association established, inside brackets.
**GWAS data**	SNPs - Genetic traits	SNP and genetic trait association trough genome wide association studies. The number of SNPs associated to each trait is presented and is followed by the p-value inside square brackets. SNPs with a p-value ≤5.0E-7 are presented in bold and green.
**Bibliography**	PubMed Link	Direct hyperlink to PubMed search of the gene in question, in human.
**Only for CNVs**	Best Hits	For CNVs or query region, the results of the overlap search, according to the preferences of the user, are presented here, in line with the beginning of the CNV. The overleaped CNV, their clinical significance, the percentage of overlap and frequency is presented. One line is presented by tested database (if any CNV falls inside the defined parameters).
