# Supplementary material for: SVInterpreter: A Comprehensive Topologically Associated Domain-Based Clinical Outcome Prediction Tool for Balanced and Unbalanced Structural Variants
Source: Front Genet. 2021 Dec 1;12:757170. doi: 10.3389/fgene.2021.757170 (PMC8671832; doi:10.3389/fgene.2021.757170)
Supplement: Supplementary file 7 [file DataSheet1.pdf]

SUPPLEMENTARY MATERIAL

Contents

1. Supplementary Methods

1.1 Output description 2

2. Step-by-step tutorial for the application of SVInterpreter, with example

2.1 Filling the input form 3
2.2 Output table interpretation 4
2.3 Example of analysis 5

3.Supplementary Figures

Supplementary Figure 1. Example of a complex rearrangement analysis 6
Supplementary Figure 2. SVInterpreter Input form overview 8

4. Supplementary Tables

Supplementary Table 1. Average TAD size by genome version and cell line or tissue 11
Supplementary Table 2. Data sources used by SVInterpreter 12
Supplementary Table 3. SVInterpreter output table column description 16
Supplementary Table 4. Distribution of the individual SVs analyzed by chromosome 19

Supplementary Table 5. (.XLSX) List of SVs analyzed with SVInterpreter on both retrospective and clinical setting, and associated data
Supplementary Table 6. (.XLSX) SVInterpreter output table example

5. References 20

Supplementary Video 1. (.MP4) Step-by-step example of the SVInterpreter form filling

1. Supplementary Methods

1.1. Output description

The output file is a XLSX file. For translocation, inversion breakpoints localized within different TADs, and insertions, the XLSX file contains two data tables, one per breakpoint or affected region. For inversions affecting only one TAD, deletions, and duplications, only one data table is presented. For CNVs, an additional sheet with the ACMG classification criteria (Riggs et al., 2020) and scores is included on the output XLSX file. Also, for CNVs, an additional XLSX file is made available on the output page, which contains the full CNV database overlap search results, following the CNV-ConTool output format (David et al., 2020).

The genomic information table contains all functional and non-functional genomic elements disrupted by the breakpoint, protein-coding, lincRNAs, lnRNAs, and functional and non-functional genomic elements with expression in GTEx database. These elements are sorted by their genomic position, intercalated with relevant intergenic regions and with the indication of the breakpoint location following the International System for Human Cytogenomic Nomenclature 2020 (McGowan-Jordan et al., 2020). For disrupted genomic elements, it is also indicated in the table, in which exon or intron the breakpoint is located. This location is identified in the corresponding transcript that produces the larger protein, according to Ensembl (Hunt et al., 2018). The intergenic regions are inserted into the table if they contain any single nucleotide polymorphism associated to a phenotypic trait or disease, according to genome wide association studies database (Buniello et al., 2019). Also, along the table, the boundaries of each TAD, and their respective identification relative to the brTAD is showed.

For each functional genomic element, several levels of information are given, divided into nine major categories:

i) genes and intergenic regions, where data directly associated to the gene function and structure is presented;

ii) clustered interactions and loops, where evidence of gene misregulation is presented;

iii) clinical phenotype, where the associated disorders and phenotypical similarity search is showed;

iv)gene fusion in cancer presents the state-of-the-art fusion genes and respective type of cancer and frequency;

v)Animal Models and associated phenotypic characteristics, where phenotypic characteristics of several knockout animal models can be found;

vi) genes associated to infertility, according to studies (Oud et al., 2019);

vii) genome-wide association studies (GWAS) results, which are presented for genes and intergenic regions; **viii) bibliography**; **ix) CNV overlap results**.

The columns that compose the nine categories are described in Supplementary Table 3.

To facilitate data interpretation, several fields of the table containing especially meaningful annotation data are automatically highlighted, including: genes included in any panel from PanelAPP (Martin et al., 2019), genes included on the actionable genes list (Kalia et al., 2017), genes with haploinsuficiency index <10% (Huang et al., 2010), triplosensitivity score=3, observed vs expected LoF variants ≤0.3 (Karczewski et al., 2020), GeneHancer cluster of interactions (Fishilevich et al., 2017) or chromatin Loops disrupted by the

breakpoint, OMIM inheritance overlapping the inputted inheritance (optional input field) and GWAS data with a p-value ≤5.0E-7.

Additionally, in the header of each table, is presented an hyperlink with the text "UCSC genome browser visualization". Through a personalized UCSC genome browser session, this link provides a graphic representation of the genomic region, helping the user with their interpretation. This session focus on the specific analyzed region, automatically marking the breakpoint or deleted/duplicated/inserted region using the highlight function from the browser. Custom tracks demarcating TADs and loops of the chosen cell-line/tissue are presented, as well as UCSC native tracks, also explored on the output table, including, among others, genes, OMIM genes, geneHancer cluster of interactions, SNPs of GWAS studies and Hi-C interactions heatmap.

2. Step-by-step tutorial for the application of SVInterpreter, with example

2.1. Filling the input form

Step 1: Access the SVInterpreter webpage, via link: https://dgrctools-insa.min-saude.pt/cgi-bin/SVInterpreter.py

Step 2: **Select the genome version to be used** (Supplementary Figure 2 A1). This must be the same version as the coordinates inputted at step 7.

Step 3: Select the Cell-line/Tissue to use as reference for the analysis (Supplementary Figure 2 A2). The reference TADs and chromatin loops comply with the Cell-line/Tissue chosen in this step. This choice must be done according to the case's characteristics. When in doubt, we suggest the use of undifferentiated cells: hESC.

Step 4: Insert phenotypic description (Supplementary Figure 2 A3). If there is phenotypic characteristics associated with the SV, they may be inserted here (optional). The characteristics must be inserted as HPO terms IDs (HPO:XXXXXXX), separated by commas. These characteristics are overlapped with the ones associated with disease by SVInterpreter.

Step 5: Select the inheritance of interest (optional) (Supplementary Figure 2 A4). If there is an specific inheritance that the user want to investigate, it can be selected here. On the output table, the diseases associated to the chosen inheritance are highlighted.

Step 6: Select the type of SV to analyze (Supplementary Figure 2 B5). The user must choose between "Balanced Translocation", "Unbalanced Translocation", "Insertion", "Deletion", "Inversion", and "Duplication", if the intention is to analyze an SV; or "Query genomic Region", if the intention is to explore a genomic region of interest. Translocations with small deletions associated to the breakpoint may be analyzed with the options "Balanced Translocation".

Step 7: Fill the chromosome and coordinates of SV to analyze (Supplementary Figure 2 B 6A,6B). Input the chromosome(s) and genomic location(s) of the SV. For translocations and insertions (6A), fields for both affected locations are opened, while for inversions, deletions, duplications, and "Query genomic region", there is only space for one region (6B). For translocations with deletions associated to the breakpoint, instead of simply inserting the breakpoint coordinate, the user may insert the deleted region (start coordinate-end coordinate). Make sure that the coordinates are in the same genome version as the one chosen on step 1.

Step 8: (CNVs only) Perform overlap search with public databases (Supplementary Figure 2 B7). For CNVs, SVInterpreter offers the option of overlapping the inputed CNV (query) with the ones available on public databases (reference). If the user selects this option, a new submenu is open, to select which databases are going to be used, and which strategy of overlap is applied: i) "mutual overlap", where both query and reference need to overlap one another by at least X percent (X also established by the user - default is 70%); ii) "query comprised by reference", where the query has to be completely contained by the reference, independently of the reference size.

Step 9: **Select if the analysis must be based on TADs or a user-defined region** (Supplementary Figure 2 C). If "TADs" option is chosen, the region to analyze is defined by the TADs not coordinates. When this option is selected, a sliding button is opened, allowing the user to define an interval of TADs to analyze (default is the TAD affected by the breakpoint: brTAD). On the other hand, if the "A specific region" option is chosen, the user is allowed to insert a genomic region (as chromosome: start-end), that must be analyzed, independently of the number of TADs it contains.

Step 10: Click on submit. This will submit the form to SVInterpreter analysis, and open the output page. This page will automatically refresh until the output table is ready for download.

2.2. Output table interpretation

Step 11: Check if any of the breakpoints disrupts/deletes/duplicates a genomic element, as a protein-coding gene, lincRNA, etc. This can be easily visualized by: i) the demarcation of the breakpoints in green, on the first column; ii) the color code of the table: red for deleted regions, blue for duplicated regions, yellow and grey to differentiate between the region upstream and downstream of the breakpoint; and iii) the indication of which Exon(s)/Intron(s) is disrupted/deleted/duplicated (if applicable) on the third column, in green. This will be the first genomic element to be investigated.

Step 12: If a gene is disrupted/deleted/duplicated, check the gene-associated information, emphasizing the following: i) dosage sensitivity values (fifth and sixth columns), according to the type of SV; ii) Gtex expression, taking special attention to high expression on tissues associated with the SV-associated phenotype (if applicable, ninth column); iii) Associated phenotypes, respective inheritance, classifications by DDG2P and ClinGen, and, if applicable, the phenotypic overlap values (12th to 15th columns); iv) Gene-phenotype/disease association in animal models, and GWAS data, with special attention to the phenotypic overlap with the SV-associated characteristics (18th to 22th and 24th columns). If there is some indication of the gene being disease-causing, one may expand the research to the bibliographic references (25th column).

Step 13: Check the brTAD genomic element content. Verify the gene-associated data for the remaining genes of the brTAD. The relevant fields are similar to step 12, adding, GeneHancer and chromatin loop disruption, which may indicate a position effect event (10th and 11th columns). Since, in this case, we are looking for indications of position effect, the evidence of gene association must be strong, as for phenotypic overlap or association with autosomal dominant diseases.

Step 14: (Specifically for CNVs) Check the overlap with public databases. On the last column of the table, on the same line of the breakpoint, the results of the overlap with

public databases is presented. This will aid the interpretation of the pathogenicity of the analyzed CNV.

Step 15: If no candidate gene was found, repeat steps 12 and 13 for flanking TADs (TAD-1 and TAD+1). This step may be repeated for the TADs further and further from the brTAD (TAD-2 and TAD+2, then TAD-3 and TAD+3, and so on) until the user feels content with their evaluation.

2.3. Example of analysis

As an example, we show the application of SVInterpreter to a translocation t(16;17) - DGRC0016. Since the input form may vary with the type of SV, other examples are showed on the tutorial available on the SVInterpreter webpage.

The filling of the form (steps 1 to 10), is illustrated in Supplementary Video 1.

Regarding the analysis of the result table (Supplementary table 6), we started by checking the disrupted genes: in the chromosome 16, ANKRD11 is disrupted at IVS2, and in chromosome 17, WNT3 gene is disrupted in IVS1. The ANKRD11 gene has a significant pLi and is associated with autosomal dominant KBG syndrome, that presents good phenotypic overlap metrics (PhenSSc 2.31 (P= 0.02 ; MaxSSc 2.91)). Also, the animal models present consistent phenotypic characteristics. At this step, ANKRD11 seems like a suitable candidate gene. WNT3 also as a significant pLi but is only associated with autosomal recessive Tetra-amilia syndrome, which shows a poor phenotypic overlap (PhenSSc 0.788 (P= 0.77 ; MaxSSc 2.91)). These and the other table information do not support the association of WNT3 with the phenotype. As for the remaining genomic elements, none presented a significant phenotypic overlap with the case, nor were associated to autosomal dominant pathologies and had their GeneHancer cluster of interactions or loops disrupted by the breakpoint. Since the disrupted gene ANKRD11 explains the totality of the phenotype, we concluded our search here. Otherwise we would extend the analysis to TADs -1 and +1.

3. Supplementary Figures

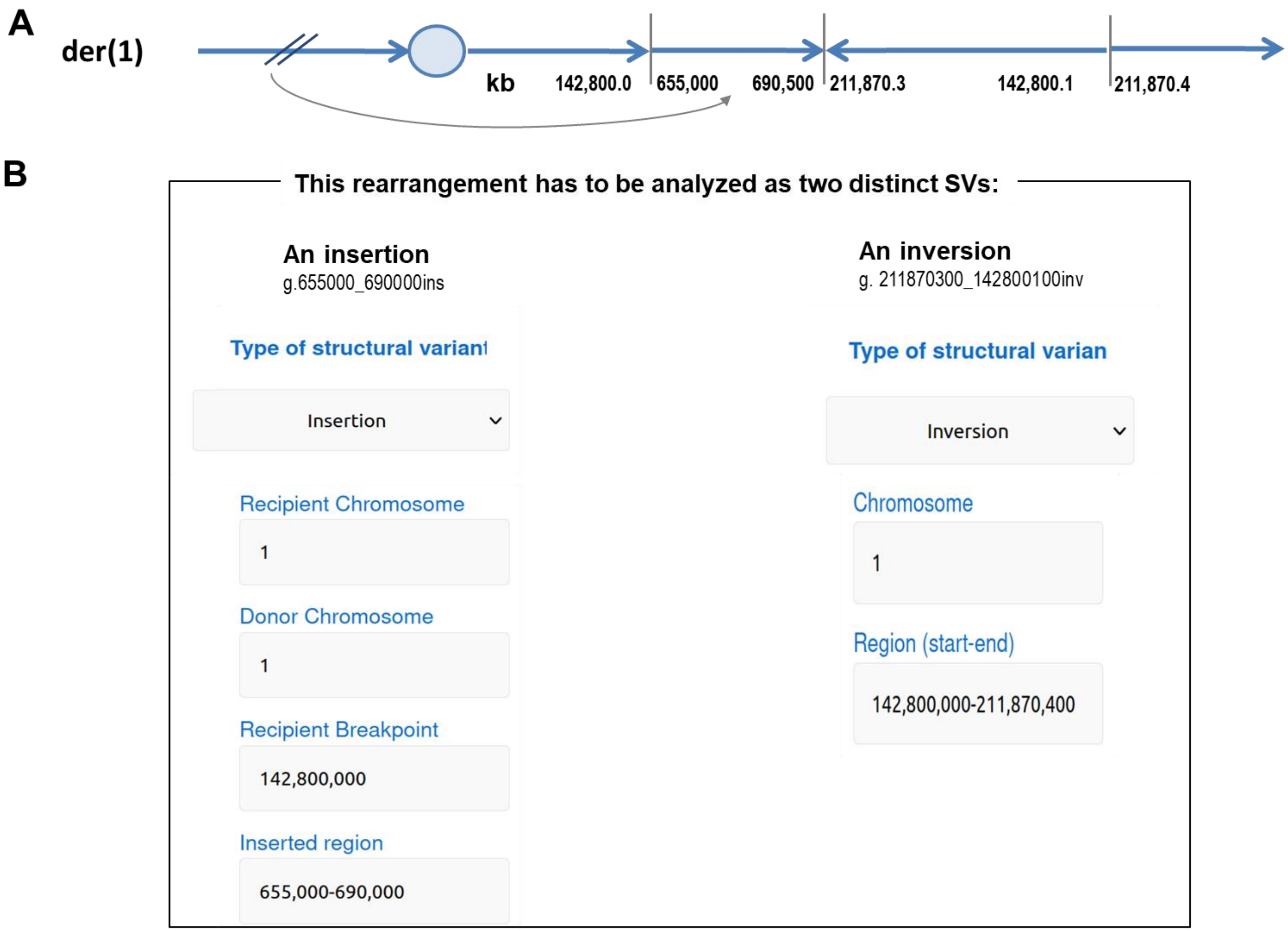

Supplementary Figure 1. Example of a complex rearrangement analysis. (A) A hypothetical complex rearrangement in chromosome 1, involves the excision and insertion of a genomic fragment from the short arm to the long arm, and an inversion. (B) For the analysis with SVInterpreter, the complex rearrangement is subdivided into an insertion and an inversion. Together, the two analyses allow a complete overview of all the regions affected by the complex rearrangement.

A

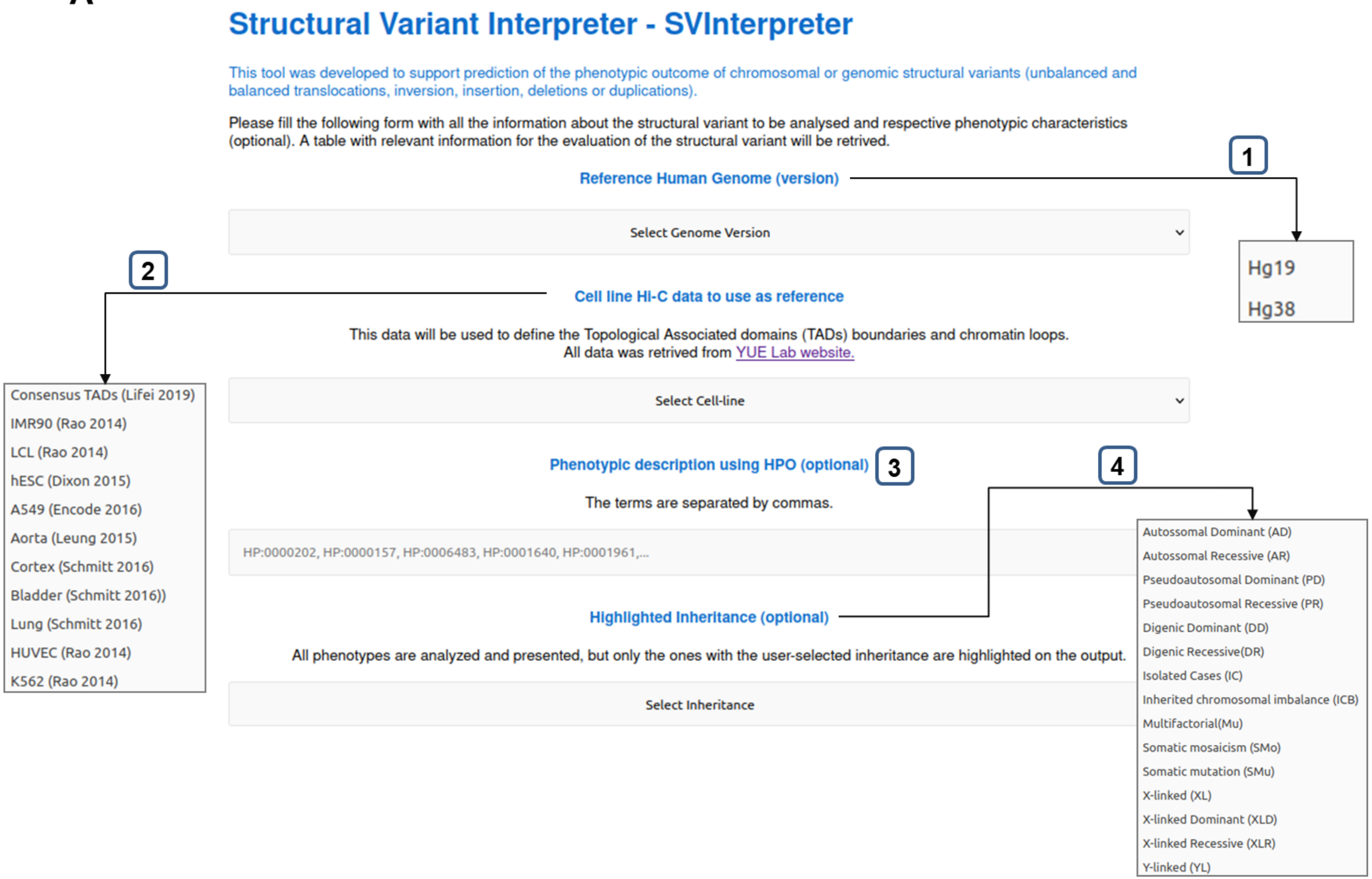
Structural Variant Interpreter - SVInterpreter
This tool was developed to support prediction of the phenotypic outcome of chromosomal or genomic structural variants (unbalanced and balanced translocations, inversion, insertion, deletions or duplications).
Please fill the following form with all the information about the structural variant to be analysed and respective phenotypic characteristics (optional). A table with relevant information for the evaluation of the structural variant will be retrived.
Reference Human Genome (version)
1
Select Genome Version
Hg19
Hg38
2
Cell line Hi-C data to use as reference
This data will be used to define the Topological Associated domains (TADs) boundaries and chromatin loops.
All data was retrived from YUE Lab website.
Consensus TADs (Lifei 2019)
IMR90 (Rao 2014)
LCL (Rao 2014)
hESC (Dixon 2015)
A549 (Encode 2016)
Aorta (Leung 2015)
Cortex (Schmitt 2016)
Bladder (Schmitt 2016))
Lung (Schmitt 2016)
HUVEC (Rao 2014)
K562 (Rao 2014)
Select Cell-line
Phenotypic description using HPO (optional)
3
4
The terms are separated by commas.
HP:0000202, HP:0000157, HP:0006483, HP:0001640, HP:0001961,...
Highlighted Inheritance (optional)
All phenotypes are analyzed and presented, but only the ones with the user-selected inheritance are highlighted on the output.
Select Inheritance
Autossomal Dominant (AD)
Autossomal Recessive (AR)
Pseudoautosomal Dominant (PD)
Pseudoautosomal Recessive (PR)
Digenic Dominant (DD)
Digenic Recessive(DR)
Isolated Cases (IC)
Inherited chromosomal imbalance (ICB)
Multifactorial(Mu)
Somatic mosaicism (SMo)
Somatic mutation (SMu)
X-linked (XL)
X-linked Dominant (XLD)
X-linked Recessive (XLR)
Y-linked (YL)

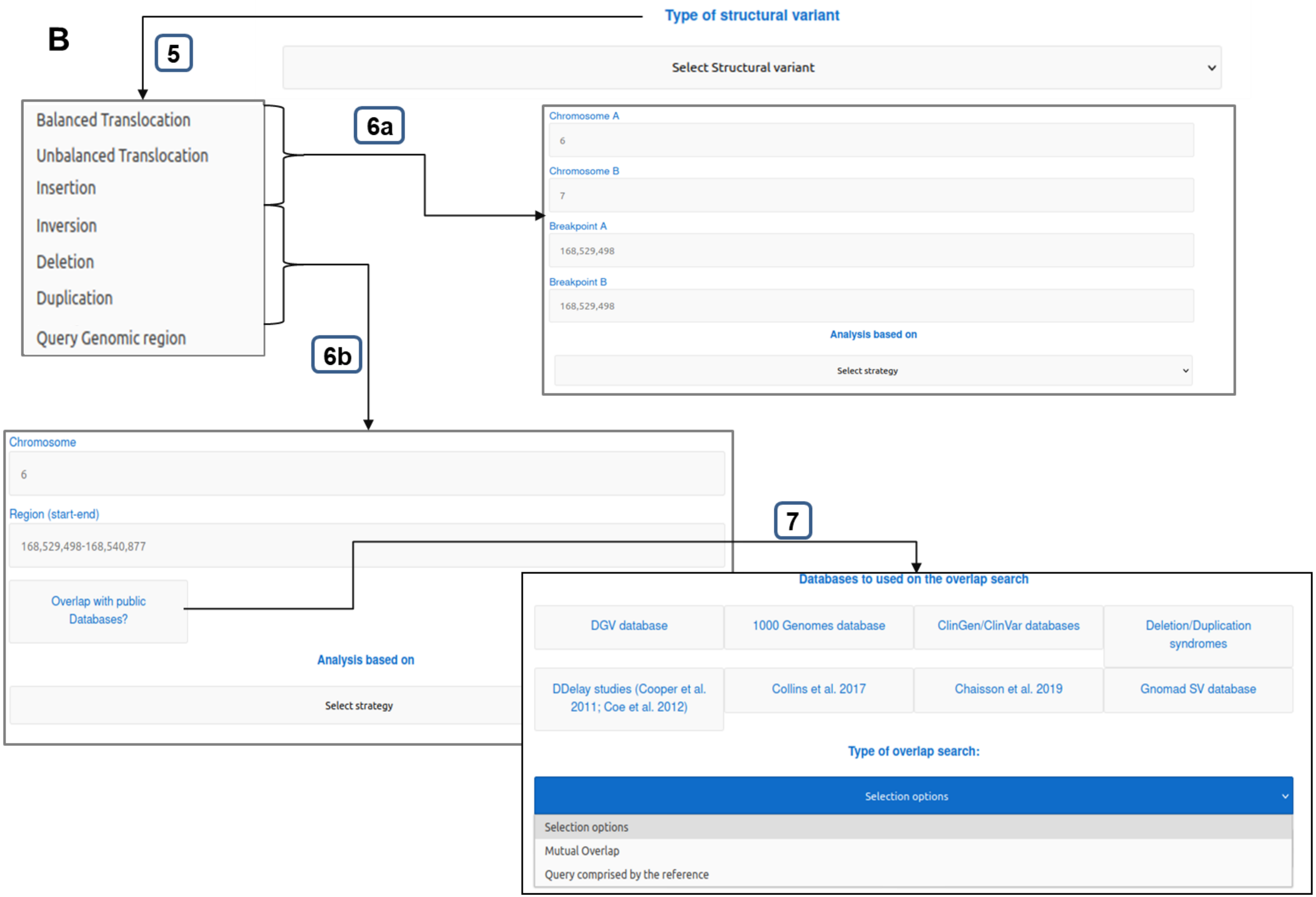

B
5
Type of structural variant
Select Structural variant
Balanced Translocation
Unbalanced Translocation
Insertion
Inversion
Deletion
Duplication
Query Genomic region
6a
Chromosome A
6
Chromosome B
7
Breakpoint A
168,529,498
Breakpoint B
168,529,498
Analysis based on
Select strategy
6b
Chromosome
6
Region (start-end)
168,529,498-168,540,877
Overlap with public Databases?
Analysis based on
Select strategy
7
Databases to used on the overlap search
DGV database
1000 Genomes database
ClinGen/ClinVar databases
Deletion/Duplication syndromes
DDelay studies (Cooper et al. 2011; Coe et al. 2012)
Collins et al. 2017
Chaisson et al. 2019
Gnomad SV database
Type of overlap search:
Selection options
Selection options
Mutual Overlap
Query comprised by the reference

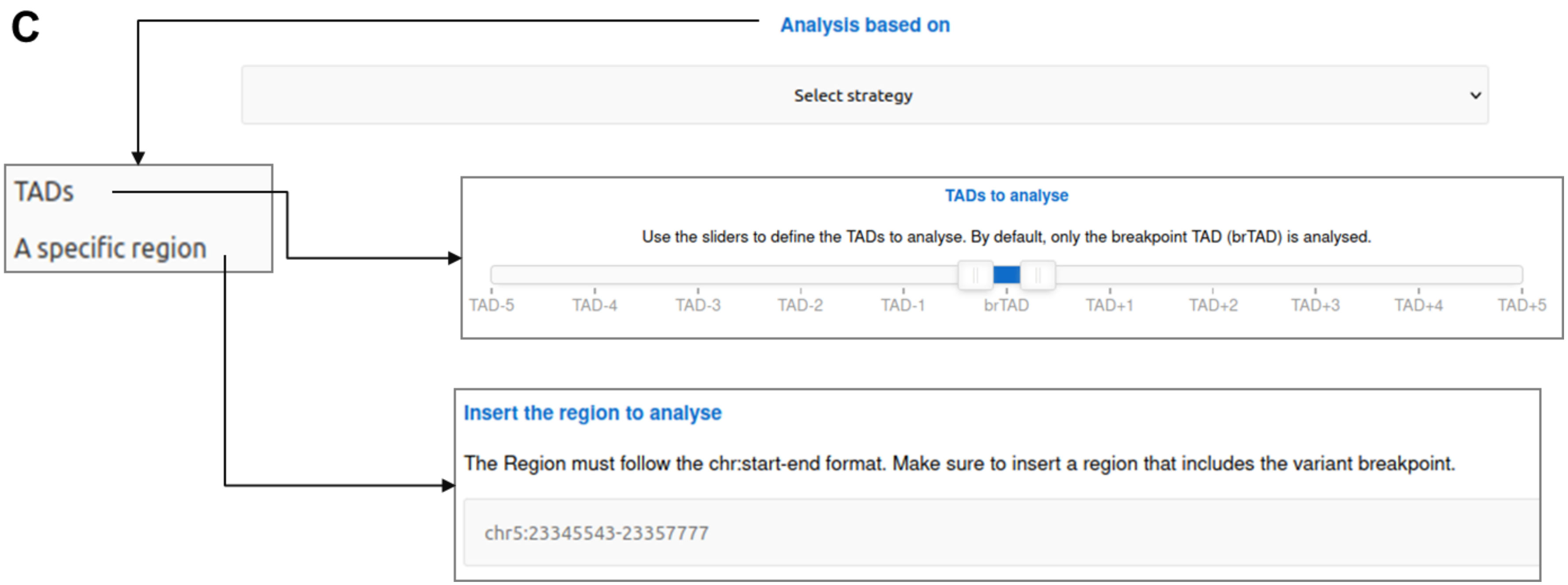

Supplementary Figure 2. SVInterpreter Input form overview. The SVInterpreter form can be divided into three parts, (**A**) the general parameters, (**B**) SV specific parameters, and (**C**) Selection of region to analyze.

(**A**) Starts with the selection of the (**1**) human genome version and the (**2**) reference cell line to be used for TAD and Loop search. Then, two optional parameters: (**3**) phenotypic description of the case in question, which must be inputted using Human Phenotype Ontology terms separated by commas; and (**4**) the selection of an inheritance of interest, where the disorders with the selected inheritance will be highlighted on the output table.

(**B**) Then, (**5**) type of variant is chosen, where each type of variant will open a slightly different form. For insertions, balanced and unbalanced translocations, the form is similar to the one showed in (**6a**), where the user sets the chromosomes and breakpoints. For deletions, duplications, and inversions a form similar to (**6b**) is showed, with the choice of CNV database overlap search, for deletions and duplications. Choosing the (**7**) CNV overlap search, the user must select which databases to use, and the type of overlap, as described in David et al., (2020).

(**C**) Lastly, the user can choose between using TADs to define the region to analyze (where the user can choose up to 5 TADs upstream (+5) or downstream (-5) the breakpoint) or set the region manually using genomic coordinates.

4. Supplementary Tables

Supplementary Table 1. Average TAD size by genome version and cell line or tissue

Cell line/Tissue	Hg38 average TAD size (bp)	Hg19 average TAD size (bp)
Consensus TADs	1,796,527	1,786,131
IMR90	858,229	824,472
LCL (GM12878)	815,201	835,946
hESC	1,087,767	1,058,135
A549	1,405,466	1,411,623
Aorta tissue	1,679,636	1,690,650
Cortex tissue	1,736,794	1,337,331
Bladder tissue	1,799,711	1,672,068
Lung tissue	1,754,399	1,435,346
HUVEC	1,072,369	923,778
K562	842,166	867,120

This average TAD size values are used to define the regions to analyze on chromosome Y. For each chromosome Y breakpoint, the region to analyze will start at [breakpoint – (average TAD size/2)] and end at [breakpoint+(average TAD size/2)]

Supplementary Table 2. Data sources used by SVInterpreter

Data	Description	Information used	Source	Reference
Topological Associated Domains (TADs)	TADs are functional units of the genome. They are self-interacting genomic regions, which means that DNA sequences inside the TAD physically interact with each other more frequently than with sequences outside the TAD.	The TADs are used as measure units for the output table construction, defining the interest regions to be analyzed on the context of a variant.	http://3dgenome.fsm.northwestern.edu/publications.html	Rao et al., 2014; Dixon et al., 2015; Lajoie et al., 2015; Leung et al., 2015; Schmitt et al., 2016; Li et al., 2019
Genomic elements (Ensembl)	Ensembl is a genome browser for vertebrate genomes that supports research in comparative genomics, evolution, sequence variation and transcriptional regulation.	Genomic elements located interest regions, and associated information, including orientation and synonyms.	https://www.ensembl.org/index.html	Hunt et al., 2018
Genecards	Database of human genes that provides concise genomic related information, on all known and predicted human genes.	The direct hyperlink for each gene on the database is made available.	https://www.genecards.org/	Stelzer et al., 2016
Genomics England PanelApp	PanelApp is a publicly available knowledgebase that allows virtual gene panels related to human disorders to be created, stored, and queried.	Indication of which categories the panel that contains the gene fits and the respective level of evidence.	https://panelapp.genomicsengland.co.uk/	Martin et al., 2019
Actionable genes	The American College of Medical Genetics and Genomics (ACMG) has compiled a list of 59 genes, for which specific mutations are known to be causative of disorders with defined phenotypes that are clinically actionable by an accepted intervention.	Indication if the gene is on the list of the 59 genes.	https://www.ncbi.nlm.nih.gov/clinvar/docs/acmg/	Kalia et al., 2017
OMIM	OMIM is a comprehensive, authoritative compendium of human genes and genetic phenotypes	OMIM gene ID, gene function description, associated	https://omim.org/	nd

		phenotypes, inheritance and HPO phenotypic characteristics.		
Haploinsufficiency index (HI)	Describes a model of dominant gene action in diploid organisms, in which a single copy of the wild-type allele at a locus in heterozygous combination with a variant allele is insufficient to produce the wild-type phenotype.	HI of each gene.	https://decipher.sanger.ac.uk/about/downloads/data	Huang et al., 2010
Triplosensitivity (Triplo)	Evidence that a duplication of the genomic region leads to a specific phenotype.	Triplo of each gene.	https://dosage.clinicalgenome.org/	nd
probability loss of function (pli) and observed vs expected ratio (oe score)	Measures the tolerance of a gene to loss of function mutation.	pli and confidence interval of oe score for each gene.	https://gnomad.broadinstitute.org/	Karczewski et al., 2020
Uniprot	Comprehensive, high-quality and freely accessible resource of protein sequence and functional information	Direct link to the set of proteins obtained from each specific gene, for human and other animal models.	https://www.uniprot.org/	Bateman et al., 2021
GTEx expression	Public resource to study tissue-specific gene expression and regulation.	Top 3 expressed tissues for each genomic elements, including their expression quantification, the mean expression and total expression.	https://www.gtexportal.org/home/	Ardlie et al., 2015
Clustered interactions of GeneHancer	GeneHancer is a database of human regulatory elements (enhancers and promoters) and their inferred target genes	Region of interaction of each gene.	https://www.genecards.org/	Fishilevich et al., 2017
Chromatin Loops	Loops of interaction specific for the cell line or tissue chosen for the TADs	Regions involved on the loop, including or not genomic elements.	http://3dgenome.fsm.northwestern.edu/publications.html	Salameh et al., 2020

Developmental disorder gene to phenotype (DDG2P)	Integrates data from genes, variants, and phenotypes regarding developmental disorders.	Phenotypes associated to genomic elements, and respective classification.	https://www.ebi.ac.uk/gene2phenotype/disclaimer	Wright et al., 2015
ClinGen	CliGen evidence of the association between a gene and a phenotype	Phenotypes associated to genomic elements, and respective classification.	https://clinicalgenome.org/	Rehm et al., 2015
HPOSim similarity analysis	Calculation of similarities between groups of HPO terms.	Similarity score calculation between case's phenotype and gene-associated phenotypes. The similarity score, Maximum score and p-value is showed for each case.	https://cran.r-project.org/src/contrib/Archive/HPOSim/	Deng et al., 2015
Fusion Gene in cancer	Data about fusion genes found in different types of cancer according to the Mitelman Database and Atlas of Genetics and Cytogenetics in Oncology and Haematology	Fusion genes involving each gene, the type of cancer and the respective number of cases.	http://atlasgeneticsoncology.org/ https://mitelmandatabase.isb-cgc.org/	Huret et al., 2013
C. elegans model organism (WormBase)	WormBase contains accurate, current, accessible information concerning the genetics, genomics and biology of *C. elegans* and related nematodes.	Orthologs of the human genes identified and respective knockout phenotypic characteristics.	https://wormbase.org	Harris et al., 2020
Drosophila model organism (FlyBase)	FlyBase contains user-friendly information concerning the biology of *Drosophila*.	Orthologs of the human genes identified and respective knockout phenotypic characteristics.	https://flybase.org/	Thurmond et al., 2019
Mouse model organism (MGI)	MGI provides integrated genetic, genomic, and biological mouse data to facilitate the study of human health and disease.	Orthologs of the human genes identified and respective knockout phenotypic characteristics.	http://www.informatics.jax.org/	Eppig, 2017

Zebrafish model organism (zfin)	Database of genetic and genomic data for zebrafish (*Danio rerio*) providing a wide array of expertly curated, organized, and cross-referenced zebrafish research data.	Orthologs of the human genes identified and respective knockout phenotypic characteristics.	https://zfin.org/	Sprague et al., 2003
Infertility genes	Genes associated to infertility	Type of infertility disorder associated to the respective gene.	nd	Oud et al., 2019
Genome wide association studies (GWAS) - SNP data	Human catalog of phenotype-associated SNPs and genes	SNPs, genes and phenotypic characteristics and the level significance according to the p-value.	https://www.ebi.ac.uk/gwas/	Buniello et al., 2019
PubMed	Gene associated publications	Automatic search of the genes in the PubMed database.	https://pubmed.ncbi.nlm.nih.gov/	nd
CNV databases	Benign to Pathogenic CNVs, from several public curated databases, including DGV, 1000 genomes, ClinGen and Gnomad SV. Reference publications data is also used.	Benign to Pathogenic CNVs, percentages of overlap, frequencies, and respective location.	http://dgv.tcag.ca/dgv/app/home https://clinicalgenome.org/ https://gnomad.broadinstitute.org/	Cooper et al., 2012; Coe et al., 2014; MacDonald et al., 2014; Rehm et al., 2015; Collins et al., 2017; Chaisson et al., 2019; Karczewski et al., 2020
Marrvel	Integration of human and model organism genetic resources to facilitate functional annotation of the human genome.	Retrieving integrated data, namely the association between human genes and their respective orthologs.	http://marrvel.org/	Wang et al., 2017

nd - Not determined

Supplementary Table 3. SVInterpreter output table column description

Category	Column	Description
**Genes and intergenic regions**	Genomic elements; Pannels from PannelApp	Genes located inside the TAD; The principal symbol of the gene is presented, with the respective synonyms (if exist) inside brackets. The gene is followed by the PanelAPP associated panels and respective level of evidence, in which case, it's all presented in bold. The gene symbol has a hyperlink to the GeneCard respective page. Intergenic regions and the breakpoint locations are also presented in this column.
	Actionable Genes (MAGs)	The American College of Medical Genetics and Genomics actionable genes are presented in this column. If exists, appears in bold.
	Breakpoint location; Genome strand	If the gene is disrupted or partially deleted/duplicated, the affected region is presented here. Then the strand of the gene is presented next, as SS for sense, and AS for antisense. Breakpoint location and strand are separated by a semicolon. If information about the disruption is present, this field appears in bold and green.
	Gene ID	Gene ID on the OMIM database. The ID has a hyperlink to the respective page on the OMIM website.
	HI% ; Triplo	Indicates the Haploinsuficiency index and triplosensitivity score of the gene, separated by a semicolon. If the Haploinsuficiency index is lower than 10% or the triplosensitivity score is equal to 3, this field appears in bold and green.
	pLi; o/e score	Indicates the probability of loss of function and the observed vs expected score, both values separated by a semicolon. If the observed vs expected score is lower than 0.3, this field appears in bold and green.
	Protein entries	Direct link to Uniprot database, to a set of proteins associated to the respective gene, in the human species.
	Function	Description of the gene function, according to OMIM. The description might not be complete since this field is limited by the number of characters allowed on a XLSX cell.
	Top 3 highest TPM (Total TPM; Mean TPM)	The three most expressed tissues according to GTEx, in Transcripts per million (TPM). Besides the value of expression of each individual tissue, the total and mean expression are also presented inside brackets and separated by a semicolon.
**Clustered interactions and Loops**	Clustered interactions	The region covered by the cluster of interactions of each gene, according to GeneHancer. If the region is disrupted by the breakpoint, the text is presented in bold and green.
	Loops	Loops identified on the cell line or tissue chosen in the input form. The two genomic regions involved on the Loop are inside brackets, preceded by the genomic elements that they affect, and separated by "&&". If the loop is disrupted by the alteration, the field is presented in bold and green.

**Clinical phenotype**	Assoc. Disorder	Description of the disorder associated to the gene. This field has the direct hyperlink to OMIM, DDG2P or ClinGen, according to the source of the information.
	OMIM_ID_inh	ID of the OMIM phenotype indicated in the previous column, and the respective inheritance separated by an underscore, with hyperlink to the respective page on the OMIM website. If the inheritance matches the one chosen by the user on the input form (optional), this field appears in bold.
	DDG2P class.	Classification of the disorders described on the Assoc. Disorder column, according to DDG2P.
	ClinGen class.	Classification of the disorders described on the Assoc. Disorder column, according to ClinGen.
	PhenSSc (P; MaxSSc)	Result of the phenotype similarity search if any phenotype was inputted on the input form (optional). The first score is the similarity score between the inputted phenotype and the disorder described on the Assoc. Disorder column. Next, inside brackets, and separated by a semicolon is the p-value that reflects the probability of this score been obtained by chance and the maximum score that could be attained with the inputted phenotype description. This search is only applicable to disorders described on OMIM, with associated phenotypic description.
**Gene fusion in cancer**	Gene1 / Gene2 Cytoband	Fusion genes found in cancer. The two genes fused are separated by a bar, and followed by the cytoband of the second gene, separated by an underscore. The first gene is always the one being described in this line. The text has hyperlink to the Atlas database or the Mitleman database.
	Organ: type, nr. Cases	Organ and the type of cancer where the previous fusion gene was found, followed by the number of cases. The text has hyperlink to the Atlas database or the Mitleman database.
**Gene-phenotype/disease associations and animal models**	C.elegans	Phenotypic characteristics of the knockout results of the Orthologs of the human gene in *C.elegans*, with link to WormBase. Also a direct link to Uniprot search in *C.elegans* is provided.
	Drosophila	Phenotypic characteristics of the knockout results of the Orthologs of the human gene in fruit fly, with link to FlyBase. Also, a direct link to Uniprot search in fruit fly is provided.
	Mouse	Phenotypic characteristics of the knockout results of the Orthologs of the human gene in mouse, with link to MGI. Also, a direct link to Uniprot search in mouse is provided.
	Rat	Phenotypic characteristics of the knockout results of the Orthologs of the human gene in rat, with link to RGD. Also a direct link to Uniprot search in rat is provided.
	Zebrafish	Phenotypic characteristics of the knockout results of the Orthologs of the human gene in zebrafish, with link to Zfin. Also, a direct link to Uniprot search in zebrafish is provided.

**Infertility**	Disorder	Infertility-associated disorders, that were potentially or confirmed as associated with the gene in question. The disorder is presented in the column, followed by the type of association established, inside brackets.
**GWAS data**	SNPs - Genetic traits	SNP and genetic trait association trough genome wide association studies. The number of SNPs associated to each trait is presented and is followed by the p-value inside square brackets. SNPs with a p-value ≤5.0E-7 are presented in bold and green.
**Bibliography**	PubMed Link	Direct hyperlink to PubMed search of the gene in question, in human.
**Only for CNVs**	Best Hits	For CNVs or query region, the results of the overlap search, according to the preferences of the user, are presented here, in line with the beginning of the CNV. The overleaped CNV, their clinical significance, the percentage of overlap and frequency is presented. One line is presented by tested database (if any CNV falls inside the defined parameters).

Supplementary Table 4. Distribution of the individual SVs analyzed by chromosome

	Chromosome																							
	**1**	**2**	**3**	**4**	**5**	**6**	**7**	**8**	**9**	**10**	**11**	**12**	**13**	**14**	**15**	**16**	**17**	**18**	**19**	**20**	**21**	**22**	**X**	**Y**
**Translocation**	15	10	10	5	10	7	9	5	6	6	11	7	5	13	4	4	8	5	6	3	0	1	8	2
**Inversion**	1	5	1	1	1	1	1	0	0	0	0	0	1	0	0	0	0	0	0	0	0	0	5	0
**Deletion**	5	8	5	1	1	2	3	1	4	3	2	1	3	3	5	6	1	1	1	2	0	0	2	0
**Duplication**	1	6	3	0	3	2	2	2	3	1	2	3	3	2	4	6	4	2	2	0	0	2	7	0
**Insertion**	1	1	3	0	0	0	0	1	1	0	0	0	0	0	0	0	1	0	0	0	0	1	0	0
**Total**	**23**	**30**	**22**	**7**	**15**	**12**	**15**	**9**	**14**	**10**	**15**	**11**	**12**	**18**	**13**	**16**	**14**	**8**	**9**	**5**	**0**	**4**	**22**	**2**

Interchromosomal SVs are accounted for in both chromosomes involved in the rearrangement, while intrachromosomal SVs are only counted once.

5. Supplementary References

Ardlie, K. G., DeLuca, D. S., Segrè, A. V., Sullivan, T. J., Young, T. R., Gelfand, E. T., et al. (2015).
The Genotype-Tissue Expression (GTEx) pilot analysis: Multitissue gene regulation in humans. *Science* 348, 648–660. doi:10.1126/science.1262110.

Bateman, A., Martin, M. J., Orchard, S., Magrane, M., Agivetova, R., Ahmad, S., et al. (2021). UniProt: The universal protein knowledgebase in 2021. *Nucleic Acids Res.* 49, D480–D489. doi:10.1093/nar/gkaa1100.

Buniello, A., Macarthur, J. a. L., Cerezo, M., Harris, L. W., Hayhurst, J., Malangone, C., et al. (2019). The NHGRI-EBI GWAS Catalog of published genome-wide association studies, targeted arrays and summary statistics 2019. *Nucleic Acids Res.* 47, D1005–D1012. doi:10.1093/nar/gky1120.

Chaisson, M. J. P., Sanders, A. D., Zhao, X., Malhotra, A., Porubsky, D., Rausch, T., et al. (2019). Multi-platform discovery of haplotype-resolved structural variation in human genomes. *Nat. Commun.* 10:1784. doi:10.1038/s41467-018-08148-z.

Coe, B. P., Witherspoon, K., Rosenfeld, J. a., Van Bon, B. W. M., Vulto-Van Silfhout, A. T., Bosco, P., et al. (2014). Refining analyses of copy number variation identifies specific genes associated with developmental delay. *Nat. Genet.* 46, 1063–1071. doi:10.1038/ng.3092.

Collins, R. L., Brand, H., Redin, C. E., Hanscom, C., Antolik, C., Stone, M. R., et al. (2017). Defining the diverse spectrum of inversions, complex structural variation, and chromothripsis in the morbid human genome. *Genome Biol.* 18:36. doi:10.1186/s13059-017-1158-6.

Cooper, G. M., Coe, B. P., Girirajan, S., Rosenfeld, J. a, Vu, T., Williams, C., et al. (2012). A Copy Number Variation Morbidity Map of Developmental Delay. *Nat. Genet.* 43, 838–846. doi:10.1038/ng.909.A.

David, D., Freixo, J. P., Fino, J., Carvalho, I., Marques, M., Cardoso, M., et al. (2020). Comprehensive clinically oriented workflow for nucleotide level resolution and interpretation in prenatal diagnosis of de novo apparently balanced chromosomal translocations in their genomic landscape. *Hum. Genet*. 139, 531–543. doi:10.1007/s00439-020-02121-x.

Deng, Y., Gao, L., Wang, B., and Guo, X. (2015). HPOSim: An R package for phenotypic similarity measure and enrichment analysis based on the human phenotype ontology. *PLoS One* 10:e0115692. doi:10.1371/journal.pone.0115692.

Dixon, J. R., Jung, I., Selvaraj, S., Shen, Y., Antosiewicz-Bourget, J. E., Lee, A. Y., et al. (2015). Chromatin architecture reorganization during stem cell differentiation. *Nature* 518, 331–336. doi:10.1038/nature14222.

Eppig, J. T. (2017). Mouse genome informatics (MGI) resource: Genetic, genomic, and biological knowledgebase for the laboratory mouse. *ILAR J.* 58, 17–41. doi:10.1093/ilar/ilx013.

Fishilevich, S., Nudel, R., Rappaport, N., Hadar, R., Plaschkes, I., Iny Stein, T., et al. (2017). GeneHancer: genome-wide integration of enhancers and target genes in GeneCards. *Database* 2017:bax028. doi:10.1093/database/bax028.

Harris, T. W., Arnaboldi, V., Cain, S., Chan, J., Chen, W. J., Cho, J., et al. (2020). WormBase: A modern Model Organism Information Resource. *Nucleic Acids Res.* 48, D762–D767. doi:10.1093/nar/gkz920.

Huang, N., Lee, I., Marcotte, E. M., and Hurles, M. E. (2010). Characterising and predicting haploinsufficiency in the human genome. *PLoS Genet.* 6:e1001154. doi:10.1371/journal.pgen.1001154.

Hunt, S. E., McLaren, W., Gil, L., Thormann, A., Schuilenburg, H., Sheppard, D., et al. (2018). Ensembl variation resources. *Database.* 2018:bay119. doi:10.1093/database/bay119.

Huret, J. L., Ahmad, M., Arsaban, M., Bernheim, A., Cigna, J., Desangles, F., et al. (2013). Atlas of genetics and cytogenetics in oncology and haematology in 2013. *Nucleic Acids Res.* 41, 920–924. doi:10.1093/nar/gks1082.

Kalia, S. S., Adelman, K., Bale, S. J., Chung, W. K., Eng, C., Evans, J. P., et al. (2017). Recommendations for reporting of secondary findings in clinical exome and genome sequencing, 2016 update (ACMG SF v2.0): A policy statement of the American College of Medical Genetics and Genomics. *Genet. Med.* 19, 249–255. doi:10.1038/gim.2016.190.

Karczewski, K. J., Francioli, L. C., Tiao, G., Cummings, B. B., Alföldi, J., Wang, Q., et al. (2020). The mutational constraint spectrum quantified from variation in 141,456 humans. *Nature* 581, 434– 443. doi:10.1038/s41586-020-2308-7.

Lajoie, B. R., Dekker, J., and Kaplan, N. (2015). The Hitchhiker's Guide to Hi-C Analysis: Practical guidelines Bryan. *Methods* 72, 65–75. doi:10.1016/j.ymeth.2014.10.031.

Leung, D., Jung, I., Rajagopal, N., Schmitt, A., Selvaraj, S., Lee, A. Y., et al. (2015). Integrative analysis of haplotype-resolved epigenomes across human tissues. *Nature* 518, 350–354. doi:10.1038/nature14217.

Li, L., Barth, N. K. H., Pilarsky, C., and Taher, L. (2019). Cancer is associated with alterations in the three-dimensional organization of the genome. *Cancers* 11:86. doi:10.3390/cancers11121886.

MacDonald, J. R., Ziman, R., Yuen, R. K. C., Feuk, L., and Scherer, S. W. (2014). The Database of Genomic Variants: A curated collection of structural variation in the human genome. *Nucleic Acids Res.* 42, D986–D992. doi:10.1093/nar/gkt958.

Martin, A. R., Williams, E., Foulger, R. E., Leigh, S., Daugherty, L. C., Niblock, O., et al. (2019). PanelApp crowdsources expert knowledge to establish consensus diagnostic gene panels.*Nat. Genet.* 51, 1560–1565. doi:10.1038/s41588-019-0528-2.

McGowan-Jordan, J., Hastings, R. J., and Moore, S. (2020). *An International System for Human Cytogenomic Nomenclature (2020)*. doi:10.1159/isbn.978-3-318-06867-2.

Oud, M. S., Volozonoka, L., Smits, R. M., Vissers, L. E. L. M., Ramos, L., and Veltman, J. A. (2019). A systematic review and standardized clinical validity assessment of male infertility genes. *Hum. Reprod.* 34, 932–941. doi:10.1093/humrep/dez022.

Rao, S. S. P., Huntley, M. H., Durand, N. C., Stamenova, E. K., Bochkov, I. D., Robinson, J. T., et al. (2014). A 3D map of the human genome at kilobase resolution reveals principles of chromatin looping. *Cell* 159, 1665–1680. doi:10.1016/j.cell.2014.11.021.

Rehm, H. L., Berg, J. S., Brooks, L. D., Bustamante, C. D., Evans, J. P., Landrum, M. J., et al. (2015). ClinGen - The clinical genome resource. *N. Engl. J. Med.* 372, 2235–2242. doi:10.1056/NEJMsr1406261.

Riggs, E. R., Andersen, E. F., Cherry, A. M., Kantarci, S., Kearney, H., Patel, A., et al. (2020). Technical standards for the interpretation and reporting of constitutional copy-number

variants: a joint consensus recommendation of the American College of Medical Genetics and Genomics (ACMG) and the Clinical Genome Resource (ClinGen). Genet. Med. 22, 245–257. doi: 10.1038/s41436-021-01150-9

Salameh, T. J., Wang, X., Song, F., Zhang, B., Wright, S. M., Khunsriraksakul, C., et al. (2020). A supervised learning framework for chromatin loop detection in genome-wide contact maps. *Nat. Commun.* 11, 1–12. doi:10.1038/s41467-020-17239-9.

Schmitt, A. D., Hu, M., Jung, I., Xu, Z., Qiu, Y., Tan, C. L., et al. (2016). A Compendium of Chromatin Contact Maps Reveals Spatially Active Regions in the Human Genome. *Cell Rep.* 17, 2042–2059. doi:10.1016/j.celrep.2016.10.061.

Sprague, J., Clements, D., Conlin, T., Edwards, P., Frazer, K., Schaper, K., et al. (2003). The Zebrafish Information Network (ZFIN): The zebrafish model organism database. *Nucleic Acids Res.* 31, 241–243. doi:10.1093/nar/gkg027.

Stelzer, G., Rosen, N., Plaschkes, I., Zimmerman, S., Twik, M., Fishilevich, S., et al. (2016). The GeneCards suite: From gene data mining to disease genome sequence analyses. *Curr. Protoc.Bioinform.* 54, 1.30.1–1.30.33. doi:10.1002/cpbi.5.

Thurmond, J., Goodman, J. L., Strelets, V. B., Attrill, H., Gramates, L. S., Marygold, S. J., et al. (2019). FlyBase 2.0: The next generation. *Nucleic Acids Res.* 47, D759–D765. doi:10.1093/nar/gky1003.

Wang, J., Al-Ouran, R., Hu, Y., Kim, S. Y., Wan, Y. W., Wangler, M. F., et al. (2017). MARRVEL: Integration of Human and Model Organism Genetic Resources to Facilitate Functional Annotation of the Human Genome. *Am. J. Hum. Genet.* 100, 843–853. doi:10.1016/j.ajhg.2017.04.010.

Wright, C. F., Fitzgerald, T. W., Jones, W. D., Clayton, S., McRae, J. F., Van Kogelenberg, M., et al. (2015). Genetic diagnosis of developmental disorders in the DDD study: A scalable analysis of genome-wide research data. *Lancet* 385, 1305–1314. doi:10.1016/S0140-6736(14)61705-0.
