# Supplementary material for: SVInterpreter: A Comprehensive Topologically Associated Domain-Based Clinical Outcome Prediction Tool for Balanced and Unbalanced Structural Variants
Source: Front Genet. 2021 Dec 1;12:757170. doi: 10.3389/fgene.2021.757170 (PMC8671832; doi:10.3389/fgene.2021.757170)
Supplement: Supplementary file 10 [file Image1.PDF]

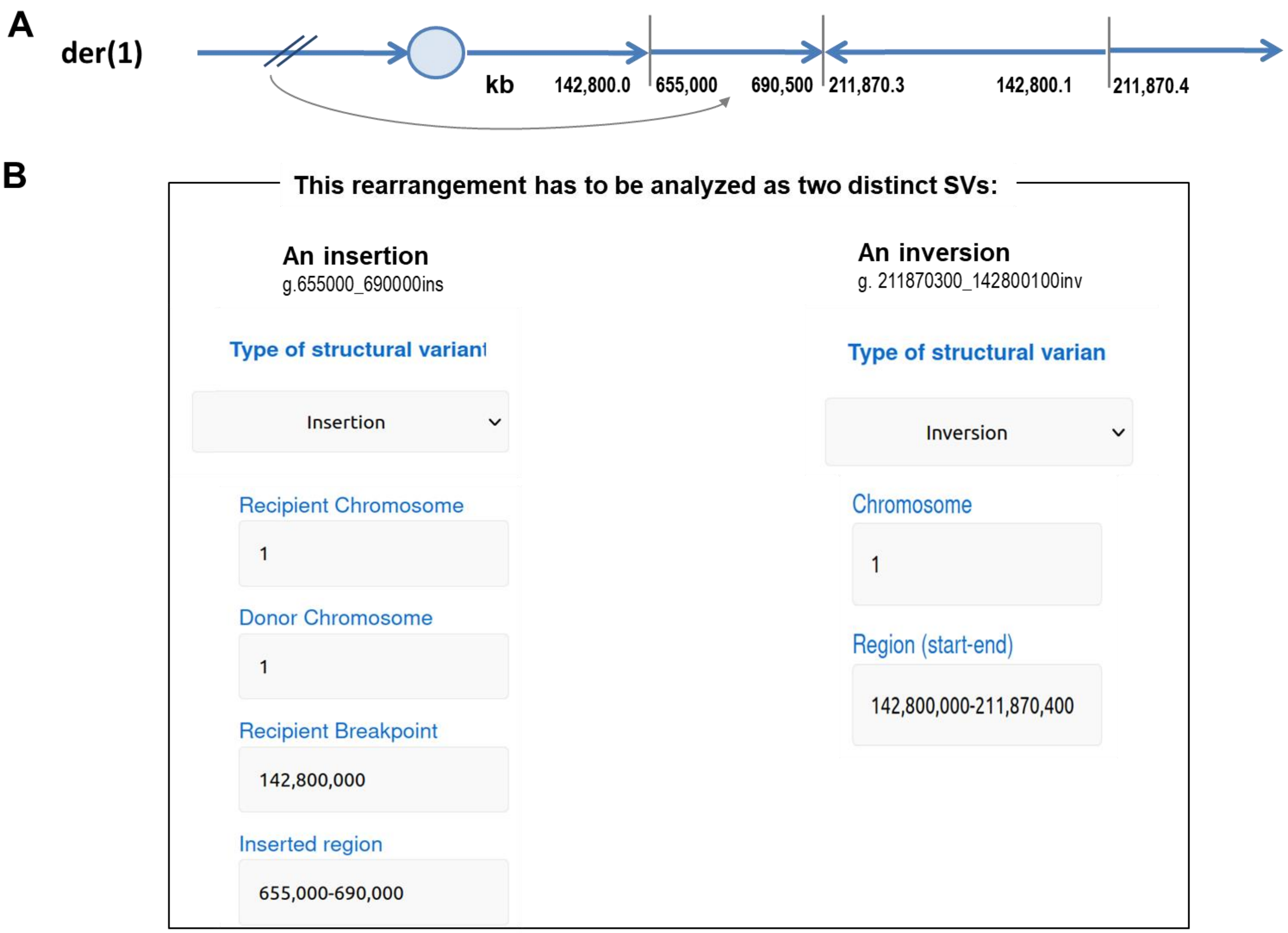
A
der(1)
kb
142,800.0
655,000
690,500
211,870.3
142,800.1
211,870.4
B
This rearrangement has to be analyzed as two distinct SVs:
An insertion
g.655000_690000ins
Type of structural variant
Insertion
Recipient Chromosome
1
Donor Chromosome
1
Recipient Breakpoint
142,800,000
Inserted region
655,000-690,000
An inversion
g. 211870300_142800100inv
Type of structural varian
Inversion
Chromosome
1
Region (start-end)
142,800,000-211,870,400

Supplementary Figure 1. Example of a complex rearrangement analysis. (A) A hypothetical complex rearrangement in chromosome 1, involves the excision and insertion of a genomic fragment from the short arm to the long arm, and an inversion. **(B)** For the analysis with SVInterpreter, the complex rearrangement is subdivided into an insertion and an inversion. Together, the two analyses allow a complete overview of all the regions affected by the complex rearrangement.
